# Icaritin Represses Autophagy to Promote Colorectal Cancer Cell Apoptosis and Sensitized Low‐Temperature Photothermal Therapy via Targeting HSP90‐TXNDC9 Interactions

**DOI:** 10.1002/advs.202412953

**Published:** 2025-04-04

**Authors:** Dan He, Siliang Chen, Xiaoyun Wang, Xiang Wen, Changyang Gong, Lei Liu, Gu He

**Affiliations:** ^1^ Division of Head & Neck Tumor Multimodality Treatment Cancer Center and Department of Dermatology & Venerology West China Hospital Sichuan University Chengdu 610041 China; ^2^ Department of Oncology The Second Affiliated Hospital of Chengdu Medical College Nuclear Industry 416 Hospital Chengdu 610051 China; ^3^ Laboratory of Dermatology Clinical Institute of Inflammation and Immunology Frontiers Science Center for Disease‐Related Molecular Network State Key Laboratory of Biotherapy West China Hospital Sichuan University Chengdu 610041 China; ^4^ Institute of Precision Drug Innovation and Cancer Center The Second Hospital of Dalian Medical University Dalian 116023 China

**Keywords:** autophagy, colorectal cancer, icaritin, photothermal therapy, HSP90

## Abstract

Colorectal cancer (CRC) ranks among the leading causes of cancer‐related dea ths worldwide, and the rising incidence and mortality of CRC underscores the urgent need for better understanding and management strategies. Icaritin (ICA) is the metabolites of icariin, a natural flavonoid glycoside compound derived from the stems and leaves of *Epimedium*. It has broad spectrum antitumor activity and inhibits the proliferation, migration, and invasion of CRC cells, and causes S phase cell cycle arrest. It exerts its antitumor effects against CRC through repressing autophagy to promote CRC cell apoptosis via interfering the HSP90‐TXNDC9 interactions. The safety and efficacy of ICA are also affirmed in a mouse xenograft model. Additionally, to test whether ICA exerts synergistic effects with low‐temperature photothermal therapy (LTPTT), a novel nanodrug delivery system, employing SiO_2_ nanocarriers, is designed aiming to load ICA with photothermal materials polydopamine (PDA), and folic acid (FA). This SiO_2_/Ica‐PDA‐FA multifunctional nanocomposite actively targets tumor tissues through the high affinity of FA for cancer cells. Once internalized, the acidic intracellular environment triggers the controlled release of ICA, inhibiting HSP90‐TXNDC9 interactions. By LTPTT and ICA drug therapy under near‐infrared illumination, a dual synergistic antitumor effect is achieved, holding promise for enhancing therapeutic outcomes in CRC treatment.

## Graphical abstract

Icaritin demonstrates broad antitumor effects by inhibiting colorectal cancer (CRC) cell proliferation, migration, invasion, and causing cell cycle arrest. It induces apoptosis by repressing autophagic flux through disrupting HSP90‐TXNDC9 interactions. Proven safe and effective in mouse models, icaritin is also integrated with low‐temperature photothermal therapy using SiO₂/PDA/FA nanocarriers for targeted, synergistic CRC treatment under near‐infrared light.

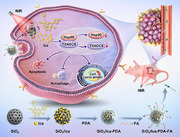

## 1 Introduction

Colorectal cancer (CRC) remains a significant global health concern, ranking among the leading causes of cancer‐related deaths worldwide. The rising incidence and mortality of CRC, particularly in individuals under 50 years of age, underscores the urgent need for better understanding and management strategies.^[^ ^1^, ^2^, ^3^, ^4^ ^]^ While surgical resection is effective in removing the majority of CRC lesions, challenges such as recurrence and metastasis persist, often occurring within a few years postsurgery.^[^ ^2^ ^]^ Additionally, for patients diagnosed with advanced‐stage disease, surgery may not be feasible. Chemotherapy and radiation therapy, though widely used, can cause significant side effects and toxicity, negatively impacting patients' quality of life. Furthermore, as treatment progresses, cancer cells may develop resistance to these therapies, adding complexity to treatment outcomes.^[^ ^3^, ^4^ ^]^ These challenges drive the need to explore novel therapeutic agents.

Icariin, a natural flavonoid glycoside compound derived from the stems and leaves of *Epimedium*, is metabolized in the body into icaritin (ICA), which exerts diverse pharmacological effects.^[^ ^5^ ^]^ In 2022, ICA capsules were approved by the China National Medical Products Administration (NMPA) for the first‐line treatment of advanced hepatocellular carcinoma (HCC), significantly improving overall survival, suggesting is that the antitumor efficacy of ICA in HCC is officially acknowledged, and the safety of ICA could be guaranteed.^[^ ^6^ ^]^ Moreover, ICA has demonstrated broad antitumor effects across various solid tumors, including CRC, prostate cancer, and breast cancer, through its ability to inhibit cancer cell proliferation.^[^ ^7^ ^]^ Therefore, ICA could serve as a promising candidate for clinical translation of CRC treatment.

Interestingly, ICA has been found to exhibit synergistic effects when combined with other antitumor therapies. Studies have shown that ICA enhances the radiosensitivity of 4T1 breast cancer cells.^[^ ^8^ ^]^ In CRC, bladder cancer, and hepatocellular carcinoma, ICA has synergized with chemotherapeutic agents, such as 5‐fluorouracil, epirubicin, and doxorubicin, respectively.^[^ ^9^, ^10^, ^11^ ^]^ Additionally, recent research suggests that ICA may synergize with immune checkpoint inhibitors in urothelial cancer,^[^ ^12^ ^]^ autophagy/mitophagy inhibitors in hepatocellular carcinoma,^[^ ^13^ ^]^ and BRD4 inhibitors like JQ1 in breast cancer.^[^ ^14^ ^]^ These findings indicate the potential of ICA to enhance therapeutic efficacy through synergistic effects across different cancer types. Since ICA is also a clinical translation candidate for CRC treatment and few previous studies have reported how to improve antitumor efficacy of ICA in CRC based on synergistic effects, elucidating the mechanism of how ICA exerting its antitumor effect in CRC is of crucial importance, which provides the theoretical basis for utilizing its synergistic effects and developing more potent therapies.

In CRC, research has revealed that ICA could mediate the opening of mitochondrial permeability transition pores in CRC cells and induce cell death by activating the c‐Jun N‐terminal kinase (JNK) pathway.^[^ ^15^ ^]^ Furthermore, ICA can induce p53 activation, leading to the suppression of AKT/mTOR signaling pathways, thereby inhibiting the proliferation of colon cancer cells.^[^ ^16^ ^]^ However, the detailed mechanism is complicated and not clear yet. In our study, we explored the mechanism of underlying the antitumor effect of ICA in CRC. Apart from inhibiting the proliferation, migration, and invasion of CRC cells, and causing cell cycle arrest in the S phase, ICA also could effectively inhibit the expression of HSP90, and interfere the interaction with its potential client protein TXNDC9 possibly through direct binding, modulating programmed cell death (PCD), mainly apoptosis and autophage, in CRC cells. Therefore, we are wondering if the inhibition of HSP90 by ICA could be utilized to design a novel synergistic therapy. HSP90 functions as a molecular chaperone protein, playing a crucial protective role within cells. In situations of extreme temperature, hypoxia, or stress induced by drugs and other factors, heat shock proteins assist in preserving protein folding and other critical structural functions.^[^ ^17^ ^]^ Studies suggest that the high expression of HSP90 in tumor cells heightens their heat resistance, consequently reducing the efficacy of photothermal therapy (PTT),^[^ ^18^ ^]^ a noninvasive strategy for tumor treatment which employs near‐infrared light‐responsive materials to convert light energy into localized hyperthermia, effectively destroying tumor cells.^[^ ^19^ ^]^ Conversely, the inhibition of HSP90 expression levels has been shown to synergistically enhance the antitumor effects of chemotherapy, radiation therapy, and other treatments.^[^ ^20^ ^]^ We further hypothesized that inhibitory effects of ICA on HSP90 might enhance the sensitivity of tumor cells to low‐temperature PTT (LTPTT), an approach which aims to achieve effective tumor ablation while mitigating the risk of tumor recurrence and metastasis associated with nonspecific heating and heat diffusion, common drawbacks of traditional PTT.

Despite its promising potential, the clinical application of ICA has been impeded by challenges, such as hydrophobic nature and low bioavailability.^[^ ^21^ ^]^ Therefore, to both utilize the inhibitory effects of ICA on HSP90 and optimize the delivery of ICA, we designed a novel nanodrug delivery system by employing SiO_2_ nanocarriers. SiO_2_ nanocarriers are one of the most promising drug delivery systems due to their excellent properties, such as dissolvability, ease of surface functionalization, biocompatibility, stability, and ease of synthesis et al. SiO_2_ nanocarriers could enhance the solubility and stability of diverse drugs without changing their physical and chemical functions, serving as a suitable carrier of ICA.^[^ ^22^, ^23^, ^24^ ^]^ In addition, we loaded ICA with photothermal materials into SiO_2_ nanocarriers, which not only improved the dispersibility of ICA in aqueous solutions but also maximized its pharmacological effects synergistically. The surface modification of SiO_2_ with polydopamine (PDA), a photothermal material, and folic acid (FA), known to enhance active targeting of tumor tissues, resulted in the development of a multifunctional nanocomposite, SiO_2_/Ica‐PDA‐FA. Upon administration, SiO2/Ica‐PDA‐FA actively targets tumor tissues through the high affinity of FA for cancer cells. Once internalized by tumor cells, the acidic intracellular environment and elevated levels of glutathione (GSH) trigger the controlled release of ICA. This, in turn, inhibits HSP90 expression within the tumor cells. This approach effectively overcomes the heat tolerance observed in tumor cells. By LTPTT and ICA drug therapy under near‐infrared (NIR) illumination, a dual synergistic antitumor effect is achieved. This dual‐action strategy holds promise for enhancing therapeutic outcomes in cancer treatment.

## 2 Results

### 2.1 ICA Inhibits the Growth, Migration, and Invasion Potential of CRC Cells

**Figure 1:**
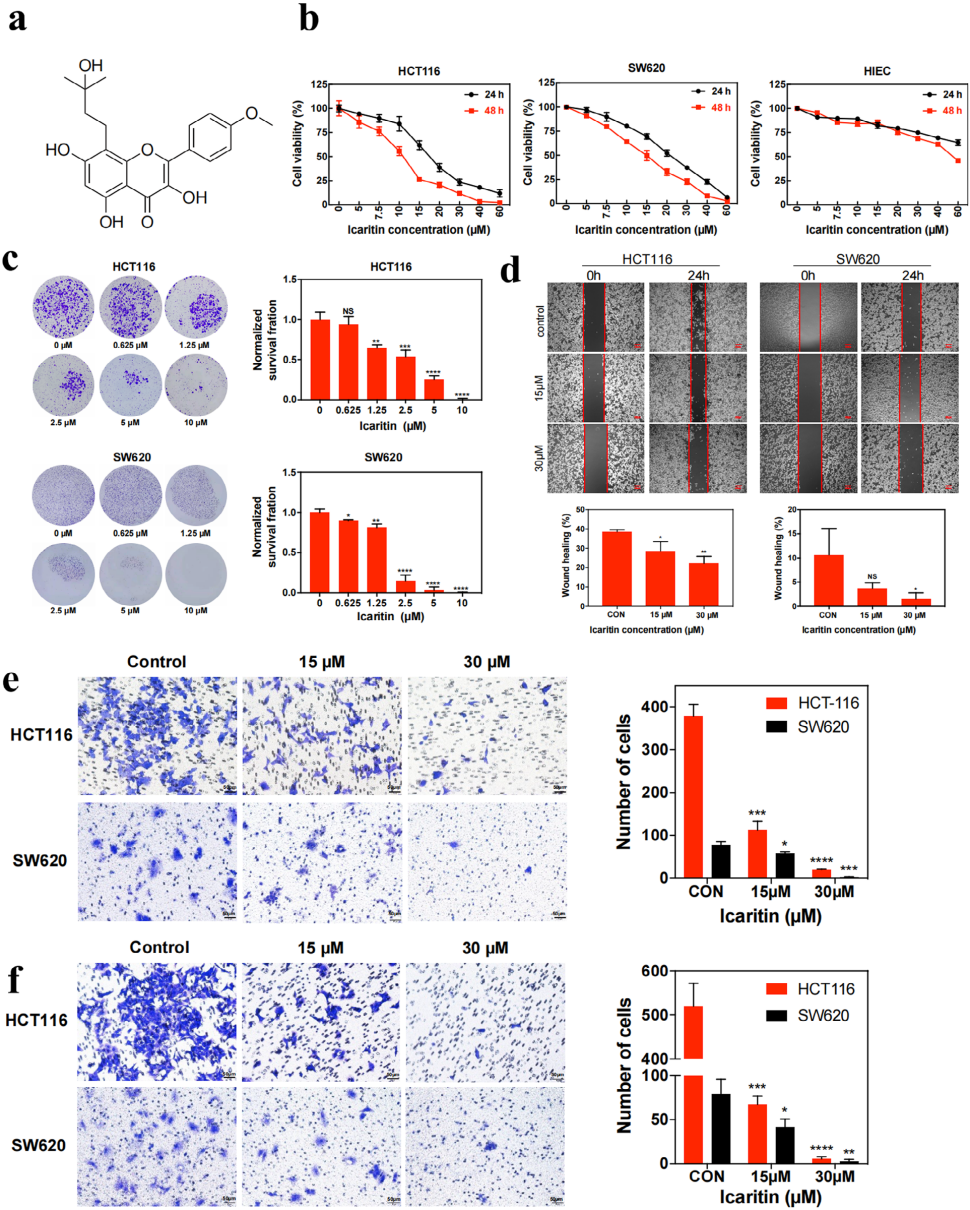
The impact of ICA on the proliferation, migration, and invasion of colorectal cancer cells. a) Chemical structure of ICA. b) The CCK‐8 assays assess the effect of ICA on the proliferation of HCT116, SW620, and the cytotoxicity of HIEC cells. c) Influence of ICA on the clonogenic ability of HCT116 and SW620 colorectal cancer cells. d) A scratch assay was used to evaluate the impact of ICA on the migration capacity of the two CRC lines. e) Transwell migration assays of HCT116 and SW620 cells upon treatment of ICA. f) Transwell invasion assays in different treatment groups of the two CRC lines. *NS*: not significant; *P < 0.05; **P < 0.01; ***P < 0.001; ****P < 0.0001.

The effect of ICA on the viability of CRC cell lines (HCT116 and SW620) and human intestinal epithelial cell lines (HIECs was determined at concentrations ranging from 0 to 60 µm for 24 and 48 h, and the results are shown in **Figure** **1**b. ICA dose‐ and time‐dependently suppressed the survival of HCT116 and SW620 cells. Specifically, after treatment with 30 µm ICA for 24 h, the inhibition rates were 75% for HCT116 cells, 50% for SW620 cells, and 25% for HIEC. However, after 48 h of treatment, ICA did not further reduce the viability of CRC cells. Nevertheless, treatment with 60 µm, ICA resulted in a 50% decrease in the viability of HIECs. Subsequent experiments were conducted using ICA at concentrations of 7.5, 15, and 30 µm.

To explore the impact of ICA on cell growth, a colony formation assay was performed. As shown in Figure 1c, the colony formation rate of both CRC cell lines was significantly lower in the ICA‐treated groups than in the negative control (NC) group. The influence of ICA on the migration and invasion was evaluated using wound scratch and Transwell assays. As depicted in Figure 1d,e, ICA treatment effectively inhibited the migration of HCT116 and SW620 cells at 30 µm in wound healing assays. However, at 15 µm, ICA had no discernible effect on the SW620 cells. In addition, the Transwell invasion assay results further confirmed this effect, with similar results (Figure 1f). In conclusion, ICA not only inhibits the proliferative capacity but also profoundly influences the migration and invasion abilities of CRC cells.

### 2.2 Bioinformatics Analysis and Identification of the Molecular Target of ICA in CRC Cells

**Figure 2:**
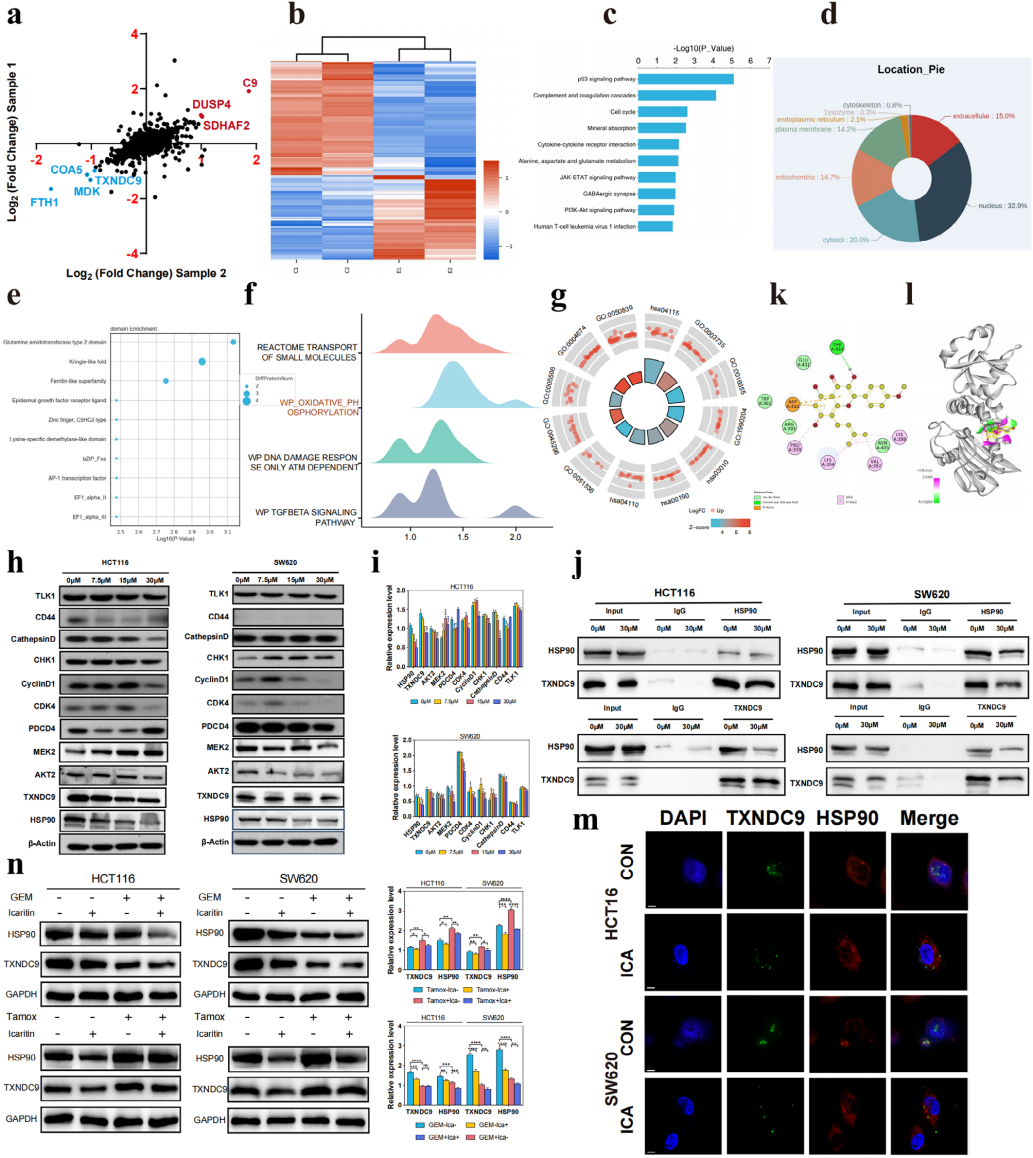
Quantitative proteomic analysis of colorectal cancer cells treated with icaritin and initial validation of the interaction between TXNDC9 and HSP90. a) Scatter plot of protein quantitative expression. b) Cluster analysis of differentially expressed proteins. c) Bar chart of significantly enriched KEGG pathways. d) Subcellular distribution of differentially expressed proteins. e) Bubble chart of significantly enriched protein structural domains. f) Mountain plot of differential protein‐encoding gene enrichment analysis. g) Circular chart of GO and KEGG pathway enrichment. h) WB analysis of differential protein expression in colorectal cancer cells treated with icaritin. i) Statistical analysis of differential protein expression levels. *P < 0.05, **P < 0.01, ***P < 0.001, ****P < 0.0001. j) Coimmunoprecipitation validation of the interaction between TXNDC9 and HSP90 in colorectal cancer HCT116 (left) and SW620 (right) cells. k) Schematic representation of the interaction plane between icaritin and the HSP90 protein. l) 3D model of the interaction between icaritin and the HSP90 protein. m) Immunofluorescence colocalization of TXNDC9 and HSP90 in CRCs. n) Western blot analysis and statistical analysis of the impact of GEM, Tamoxifen, or/and ICA on the expression of the HSP90 and TXNDC9 proteins in HCT116 (left) and SW620 (right) CRCs. *P < 0.05, **P < 0.01, ***P < 0.001, ****P < 0.0001.

To determine the underlying mechanism by which ICA impedes the progression of CRC cells, we performed a with quantitative proteomic analysis. After ICA treatment, 380 proteins were altered in HCT116 cells, comprising 161 upregulated and 219 downregulated proteins. Notably, proteins implicated in vital cell processes, such as apoptosis, the cell cycle, and autophagy, included TLK1, CDK4, PDCD4, and TXNDC9. Scatter plots (**Figure** **2**a) showing the quantitative shifts in protein expression between the control and ICA‐treated groups are shown. Particularly discernible were the significant upregulation of the C9, DUSP4, and SDHAF2 proteins, which was juxtaposed with the marked downregulation of the FTH1, COA5, MDK, and TXDNC9 proteins. Hierarchical clustering analysis of these differentially expressed proteins revealed distinct clustering between ICA‐treated and control cells, corroborated by a heatmap indicating significant differences (Figure 2b).

Kyoto Encyclopedia of Genes and Genomes (KEGG) analysis revealed enrichment of relevant pathways, including the P53 signaling pathway, complement and coagulation cascades, cell cycle, and mineral absorption (Figure 2c). Functional enrichment analysis revealed the cellular localization of these proteins, as shown in Figure 2d; these proteins spanned the cell nucleus, cytosol, extracellular space, mitochondria, and plasma membrane, with minor proportions within the endoplasmic reticulum, lysozymes, and cytoskeleton. Moreover, functional domain analysis revealed significant enrichment of domains, such as the glutamine amido‐transferase type 2 domain, Kringle‐like fold, and ferritin‐like superfamily (Figure 2e). Figure 2f shows that the differentially expressed proteins were significantly enriched in the transport of small molecules, oxidative phosphorylation, the DNA damage response, and the TGF‐β signaling pathway. Functional annotation (Figure 2g) highlighted enrichment in biological processes (BP), cellular components (CC), and molecular functions (MF), as inferred from Gene Ontology (GO) and KEGG analyses. Additionally, Western blot analysis was utilized to validate the expression patterns of proteins that were differentially expressed, as well as their upstream and downstream regulatory molecules. Consistent with the proteomic results, there was a notable decrease in the expression of TXNDC9 following ICA treatment in HCT116 cells, and similar results were observed in SW620 cells, which exhibited a concentration‐dependent decrease in HSP90 expression, as depicted in Figure 2h,i. To explore the possible interaction between HSP90 and TXNDC9 in CRC cells, co‐IP experiments were performed using an HSP90 antibody or a TXNDC9 antibody. The HSP90 antibody pulled down the TXNDC9 protein in HCT116 and SW620 cells; reciprocally, the TXNDC9 antibody captured HSP90. These results strongly indicated that these two proteins interact in CRC cells (Figure 2j). Furthermore, the influence of ICA on the interaction between HSP90 and TXNDC9 was investigated. Following treatment with 30 µm ICA, the proteins expression of HSP90 and TXNDC9 in the complexes captured by their respective antibodies was examined. These findings indicated that exposure to 30 µm ICA led to a reduction in the interaction between the TXNDC9 protein and the HSP90 protein.

To investigate the potential binding configuration between ICA and HSP90, in silico molecular docking studies were carried out (Figure 2l). ICA displayed a specific affinity for the conserved ATP binding pocket of HSP90, which is one of the known major binding sites for HSP90 inhibitors.^[^ ^25^ ^]^ Figure 2k shows that ICA bound to the active sites of the HSP90 residues: the enol hydroxyl group of ICA formed stable hydrogen bonds (green dashed lines) with THR433, while the two aromatic rings of the naphthoquinone might participate in π‐anion interactions (orange dashed lines) with ASP432. The isopentyl substituent on the naphthoquinone ring could also engage in hydrophobic interactions (pink dashed lines) with nonpolar amino acids in the vicinity, such as PRO359 and VAL397. These interactions effectively explain the selectivity of ICA for HSP90 and lay the foundation for further research into the molecular mechanisms of ICA. Additionally, dual immunofluorescence staining of HSP90 (red) and TXNDC9 (green) showed clear evidence of colocalization in CRC cells. In control cells, significant colocalization of HSP90 and TXNDC9 fluorescence signals was observed in a diffuse pattern around the nuclei. After 24 h of treatment with 30 µm ICA, the intensity of yellow fluorescence decreased, indicating that ICA interfered with the interaction between TXNDC9 and HSP90 (Figure 2m). We have also investigated the impact of the Hsp90 inhibitor GEM and the Hsp90 agonist Tamoxifen (Tamox) on Ica‐induced apoptosis and autophagy. The results demonstrated that both GEM and Ica effectively induced apoptosis in colon cancer cells, whereas Tamox only induced apoptosis to a lesser extent. Additionally, Tamox was able to partially reverse the apoptosis induced by Ica. Regarding autophagy, GEM and Ica were both capable of significantly promoting the formation of autophagic vesicles in colon cancer cells. In contrast, Tamox had an insignificant effect on autophagy induction. Notably, Ica resulted in a greater number of autolysosomes (red dot) compared to GEM. When colon cancer cells were treated concurrently with both GEM and Ica, the autophagic flux was nearly halted at the autolysosome stage, cotreatment with TAM and Ica also led to the simultaneous presence of autophagosomes (yellow dots) and autolysosomes (red dots), with the proportion of autolysosomes being lower than in the group treated solely with Ica (Figure S1, Supporting Information). These results aligned with the earlier co‐IP findings. Finally, to evaluate whether TXNDC9 is a client protein of HSP90, we treated CRC cells with the HSP90 agonist Tamox and the HSP90 inhibitor geldanamycin (GEM) in combination with ICA. As shown in Figure 2n, the addition of ICA, GEM, or a combination of both reduced the protein expression levels of HSP90 and TXNDC9. Particularly, the group treated with the combination of ICA and GEM showed notably decreased levels of HSP90 and TXNDC9 protein expression compared to the groups treated with each compound individually (*P* < 0.05). In contrast, the use of Tamox alone in CRC cells significantly increased the HSP90 and TXNDC9 protein levels, while cotreatment with ICA and Tamox led to a slight decrease in the HSP90 and TXNDC9 expression levels compared to those in the group treated with Tamox alone. Based on these experimental results, we hypothesized that TXNDC9 might be a client protein of HSP90 and that the expression levels of HSP90 directly affect the stability of TXNDC9. ICA regulated the expression of TXNDC9 by modulating HSP90.

### 2.3 ICA Suppresses Cell Proliferation in CRC Cells by Inducing Apoptosis

**Figure 3:**
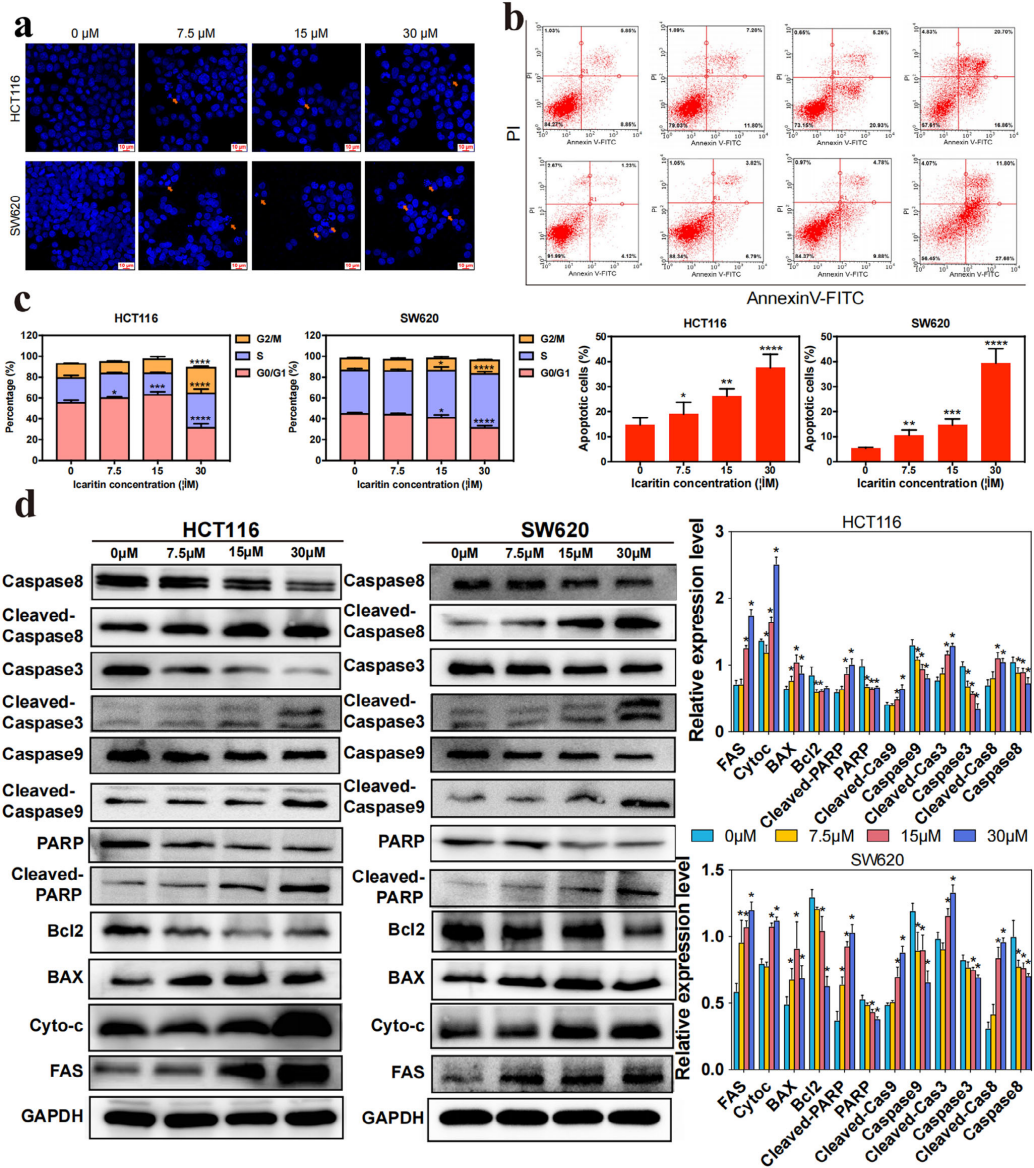
ICA induces apoptosis in colorectal cancer cells and the underlying mechanism. a) Fluorescence microscopic observation of HCT116 and SW620 cells apoptosis after ICA treatment. b) Flow cytometry analysis of the effects of ICA on the apoptosis of the two CRC lines. c) Flow cytometry analysis of the effects of ICA on the cell cycle of the two CRC lines. d) Western blot analysis of the expression of apoptosis‐related proteins in CRCs after treatment with different concentrations of ICA. *P < 0.05, **P < 0.01, ***P < 0.001, ****P < 0.0001.

To investigate the mechanism by which ICA inhibits cell proliferation, Hoechst staining was performed (**Figure** **3**a). The results showed that exposing HCT116 and SW620 cells to different concentrations of ICA led to alterations in nuclear morphology. The observed changes in karyopyknotic and nuclear fragmentation suggested an increase in the proportion of apoptotic cells with increasing concentrations of ICA in CRC cells. Additionally, apoptotic cells were assessed through Annexin V‐FITC/PI staining. Flow cytometry analysis showed that ICA significantly induced apoptosis, and as the ICA concentration increased, both the number of early (Annexin V^+^/PI^−^) and late (Annexin V^+^/PI^+^) apoptotic cells significantly increased in a dose‐dependent manner compared to that of the control (ICA 0 µm) (Figure 3b). Furthermore, the cell cycle is a crucial effector of tumor cell proliferation. The findings from flow cytometry showed an increase in the proportion of cells in the S phase compared to that in the NC group (Figure 3c). Based on these results, it was deduced that ICA can suppress the proliferation of CRC cells by regulating both apoptosis and cell cycle distribution.

To further elucidate the mechanism by which ICA triggered apoptosis in CRC cells, Western blot experiments were conducted to evaluate the activation of caspase 3, caspase 8, and caspase 9, as well as that of PARP and other proteins associated with apoptosis. The caspase family plays a crucial role in the programmed cell death process. The cascade reaction triggered by caspases is a central step in apoptosis, leading to the cleavage of specific substrates, the regulation of ribonuclease activity, and various effects, such as disruptions in cell structure, chromatin condensation, and impaired DNA repair. Finally, this cascade results in the induction of apoptosis.^[^ ^26^ ^]^ As shown in Figure 3d, the protein levels of Caspase 3, Caspase 8, and Caspase 9 decreased with increasing concentrations of ICA, whereas the proteins levels of activated Cleaved‐caspase 3, Cleaved‐caspase 8, and Cleaved‐caspase 9 increased in both CRC cell lines. These findings indicated that when ICA was applied to CRC cells, it activated initiator caspases, such as Caspase 8 and Caspase 9, initiating the cascade of caspase responses. Subsequently, it stimulates the downstream executioner caspase 3, leading to the direct cleavage of protein substrates and the induction of apoptosis in cells.^[^ ^27^ ^]^ Additionally, poly (ADP‐ribose) polymerase (PARP), a substrate of caspase, exhibited a notable increase in the ratio of cleaved PARP/PARP. The expression of the antiapoptotic protein Bcl‐2 decreased in a dose‐dependent manner, while the levels of proapoptotic protein BAX increased in a dose‐dependent manner. Furthermore, the expression levels of the mitochondrial proteins Cyto‐c and FAS trended to increase with increasing concentrations of ICA. In summary, ICA activated both the external death receptor pathway and the internal mitochondrial pathway, thereby inducing apoptosis in HCT116 and SW620 cells.

### 2.4 ICA Promotes the Formation of Autophagosomes and Inhibits Autophagic Flux in CRC Cells

**Figure 4:**
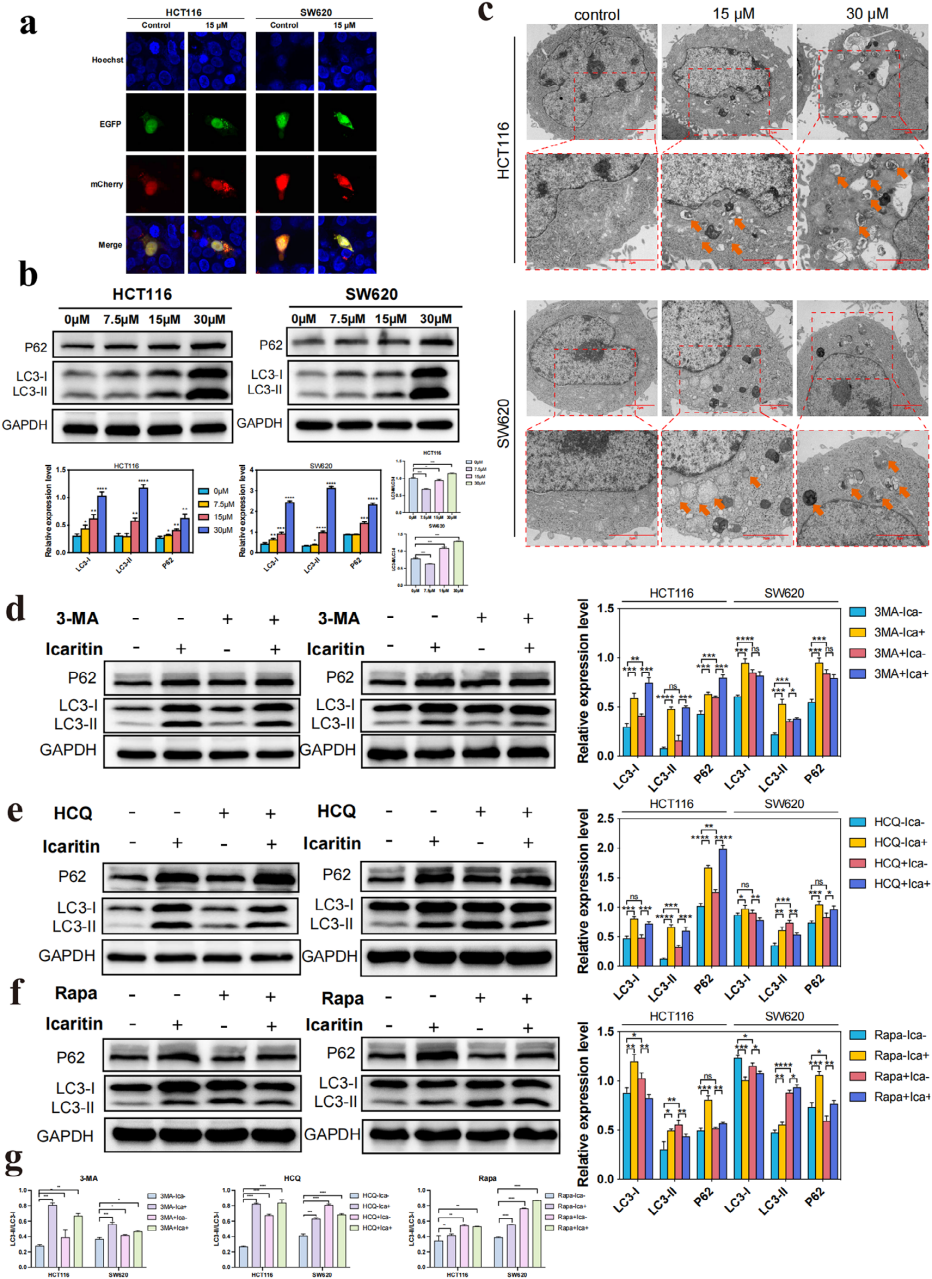
Impact of icaritin on autophagy in colorectal cancer cells. a) Confocal microscopy images of HCT116 and SW620 colorectal cancer cells transfected with the mCherry‐EGFP‐LC3B plasmid. b) Western blot analysis of the effect of icaritin on the expression of autophagy‐related proteins (P62 and LC3) in colorectal cancer cells. *P < 0.05, **P < 0.01, ***P < 0.001, ****P < 0.0001. c) Transmission electron microscopy images showing autophagosomes (orange arrows) in HCT116 and SW620 cells treated with icaritin. d) Western blot and relative expression level analysis of LC3 and P62 expression in CRCs treated with ICA combined with the autophagy inhibitor, 3‐MA. e) Western blot and relative expression level analysis of LC3 and P62 expression ICA combined with the autophagy inhibitor, HCQ. f) Western blot and relative expression level analysis of LC3 and P62 expression in CRCs treated with ICA combined with the autophagy inducer, Rapa. g) The ratio of LC3‐II/LC3‐I in d), e) and f). *P < 0.05, **P < 0.01, ***P < 0.001, ****P < 0.0001.

To explore the influence of ICA on autophagy in CRC cells, we introduced mCherry‐EGFP‐LC3B plasmids into CRC cells to evaluate cellular autophagy. During autophagy, mCherry‐EGFP‐LC3B accumulates on the autophagosome membrane, appearing as yellow puncta. Upon the fusion of autophagosomes with lysosomes, EGFP fluorescence is quenched, resulting in red puncta, which is indicative of autophagic flux.^[^ ^28^ ^]^ As shown in **Figure** **4**a, treatment with 15 µm ICA led to a notable increase in the number of yellow puncta in both cell types, suggesting that ICA likely promoted the formation of autophagosomes in CRC cells. Next, Western blot (WB) experiments were conducted to further explore the impact of ICA on autophagy‐related protein expression in CRC cells, as depicted in Figure 4b. With increasing ICA concentration, the expression levels of the autophagy‐related proteins LC3I, LC3II, and P62 increased. Throughout the autophagic process, P62 interacts with ubiquitinated proteins, forming complexes with LC3‐II proteins located on the autophagosome membrane, which are subsequently degraded within autolysosomes. The significant increase in LC3 protein expression indicated that ICA promoted autophagosome formation. On the other hand, the elevated expression of P62 suggested that ICA inhibited the degradative function of autolysosomes. Next, we proceeded to directly observe the development of autophagosomes in CRC cells subjected to ICA treatment using transmission electron microscope (TEM). Autophagosomes are characterized by double or multilayer membrane‐bound vesicular structures containing cytoplasmic components, such as mitochondria, the endoplasmic reticulum, ribosomes, etc. In contrast, autolysosomes are characterized by single‐layer membranes and are responsible for degrading cellular materials.^[^ ^29^ ^]^ Figure 4c shows that an increase in ICA concentration led to a substantial increase in the number of autophagosomes in both cell types (indicated by orange arrows). In summary, we hypothesized that ICA enhances the formation of autophagosomes in CRC cells while impeding the progression of autophagic flux. We also examined the effect of 3‐Methyladenine (3‐MA), hydroxychloroquine (HCQ), or rapamycin (Rapa) combined with ICA on autophagy in CRC cells. 3‐MA significantly reduces the LC3II/I ratio by inhibiting class III PI3K and, as previously reported.^[^ ^30^ ^]^ Immunoblot analysis revealed that cotreatment with 3‐MA and ICA resulted in an increase in the LC3II/I ratio compared to that in the group treated with 3‐MA alone, although the ratio was slightly lower than that in the group treated with ICA alone (Figure 4d). Compared to the group treated with ICA, the coadministrered 3‐MA exhibited increased p62 protein expression, indicating the inhibition of cellular autophagy. This confirmed the ability of ICA to promote autophagosome formation. To demonstrate the impact of ICA on autolysosomes in CRC cells, hydroxychloroquine (HCQ) was used. HCQ inhibits autophagolysosomal degradation by disrupting lysosomal acidification, thus halting the breakdown of the P62 protein. Intriguingly, when CRC cells were cotreated with both HCQ and ICA, there was a notable increase in the expression level of P62 compared to that in cells treated with ICA or HCQ alone (Figure 4e), confirming that ICA inhibited the degradative function of autolysosomes. Furthermore, to confirm whether ICA‐induced autophagy was potentiated by rapamycin‐induced autophagy, we observed that when rapamycin and ICA were used simultaneously, more LC3‐I was converted to LC3‐II, and p62 increased (Figure 4f,g) in comparison to that in the groups treated with rapamycin or ICA alone. Taken together, these results suggested that ICA facilitated the formation of autophagosomes while suppressing the progression of autophagic flux in CRC cells.

### 2.5 ICA Regulates Autophagy and Apoptosis in CRC Cells by Attenuating the Interaction between HSP90 and TXNDC9

**Figure 5:**
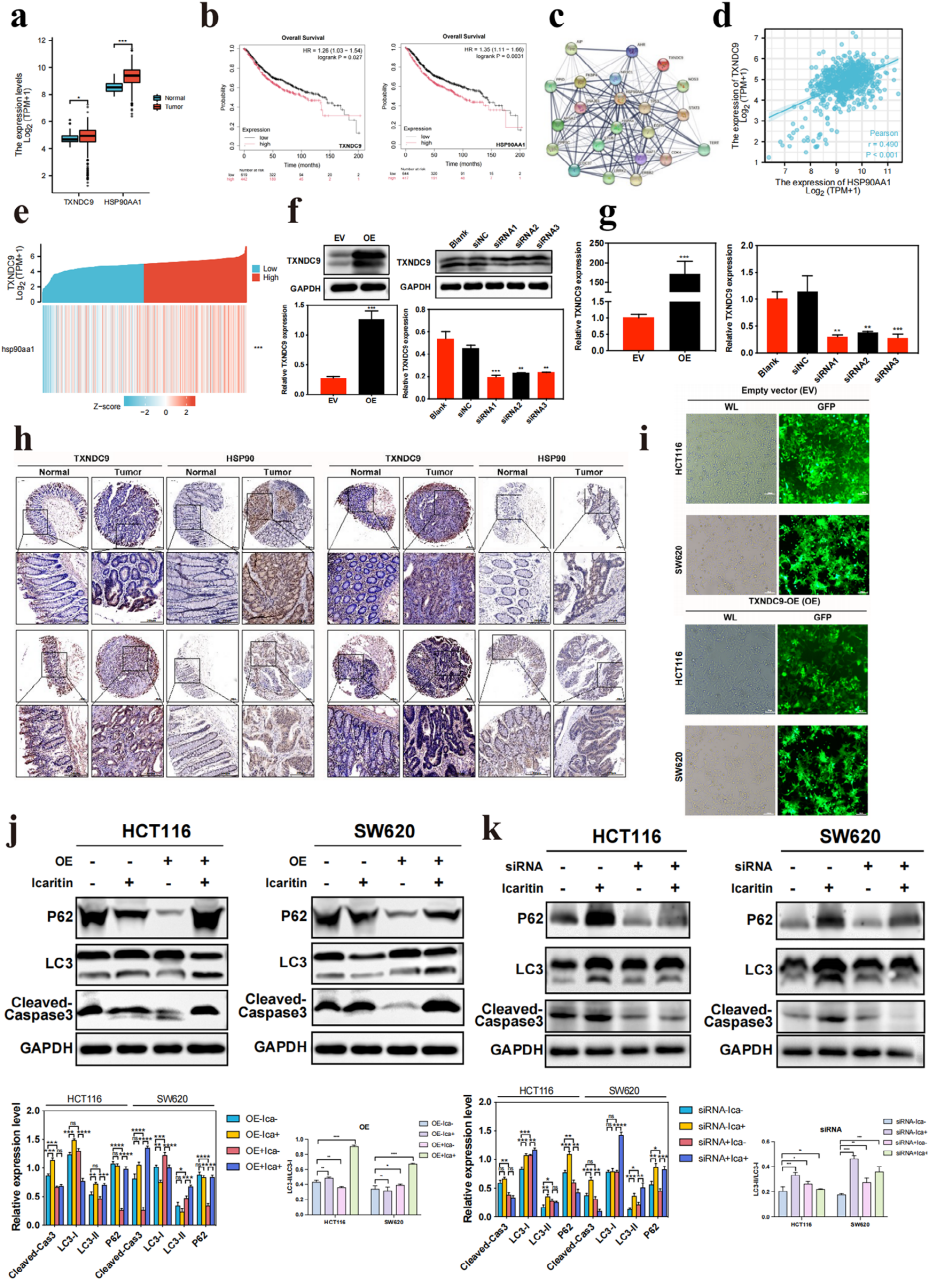
Icaritin regulates colorectal cancer cell programmed cell death by suppressing the interaction between TXNDC9 and HSP90. a) Differential expression of TXNDC9 and HSP90AA1 in colorectal cancer and adjacent tissues. b) The Kaplan–Meier plot showed that patients with up‐regulated TXNDC9 or HSP90AA1 had compromised survival. c) Interaction network between TXNDC9 and HSP90. d) Analysis of the correlation between TXNDC9 and HSP90 expression. e) Coexpression heatmap of TXNDC9 and HSP90. f) Western Blot analysis of TXNDC9 expression in CRCs overexpressing the TXNDC9 plasmid, and low TXNDC9 expression after siRNA transfection. g) qRT‐PCR analysis was performed to evaluate the expression levels of TXNDC9 in CRC cells after overexpression and knockdown. h) Immunohistochemical staining of TXNDC9 and HSP90 in a colorectal cancer tissue microarray. Scale bar: 200 µm. i) Fluorescence microscopy observation of CRCs overexpressing the TXNDC9 plasmid. j),k) Western blot analysis of apoptosis and autophagy‐related protein expression in CRCs overexpressing TXNDC9 and with low TXNDC9 expression. Quantitative analysis of the impact of different TXNDC9 expression levels on the expression of apoptosis‐ and autophagy‐related proteins in CRCs. *P < 0.05, **P < 0.01, ***P < 0.001, ****P < 0.0001.

We then examined the TXNDC9 and HSP90AA1 expression levels among CRC patients utilizing comprehensive resources from The Cancer Genome Atlas (TCGA) terminal deoxynucleotidyl transferase dUTP nick end labeling database. The results indicated markedly elevated levels of TXNDC9 and HSP90AA1 expression in the cancer tissues of CRC patients compared to their adjacent noncancerous tissues (**Figure** **5**a). The Kaplan–Meier plot generated from Kaplan–Meier Plotter, an online tool based on transcriptomic and clinical data from the Gene Expression Omnibus (GEO) database,^[^ ^31^ ^]^ showed that patients with up‐regulated TXNDC9 or HSP90AA1 had compromised survival (Figure 5b). To explore the mechanism by which ICA inhibits TXNDC9 expression and to determine whether ICA exerts its antitumor effects by targeting TXNDC9, we conducted a bioinformatics analysis of proteins potentially interacting with TXNDC9. The results revealed an interaction between TXNDC9 and HSP90AA1 (Figure 5c), with the expression level of HSP90AA1 exhibiting a positive correlation with that of TXNDC9 (Figure 5d). Trend analysis of HSP90 and TXNDC9 expression in individual CRC samples within the TCGA database demonstrated highly similar expression patterns in the cancer tissues of CRC patients (Figure 5e). Furthermore, we performed immunohistochemical (IHC) staining of CRC tissues using tissue microarrays to assess the expression of TXNDC9 and HSP90. As shown in Figure 5h, TXNDC9 and HSP90 exhibited decreased expression in adjacent noncancerous tissues. Conversely, in CRC tissues, there was a greater proportion of positive cells (brown staining), indicative of high expression of TXNDC9 and HSP90. However, further experimental validation is required to determine whether ICA functions in its antitumor effect by impeding the interaction between TXNDC9 and HSP90, consequently reducing the stability of TXNDC9.

To confirm the critical role of TXNDC9 in mediating the effects of ICA on programmed cell death in CRC cells, lentiviral vectors were constructed to overexpress or knock down TXNDC9. Figure 5i shows successful overexpression (OE) of TXNDC9 in CRC cells after infection. Western blot and qRT‐PCR analysis revealed a substantial increase in the TXNDC9 protein level following infection in the OE group, and the TXNDC9 level was decreased in the siRNAs‐transfected cells (***P < 0.001) (Figure 5f). siRNA1 was chosen for further experiments. Additionally, to confirm whether ICA regulated autophagy and apoptosis through TXNDC9, the levels of the autophagy‐associated proteins P62 and LC3, as well as the apoptosis‐related protein cleaved caspase 3, were assessed in CRC cells with overexpression TXNDC9. As shown in Figure 5j, compared with untreated CRC cells, CRC cells with elevated TXNDC9 expression displayed increased LC3I expression and reduced LC3II, P62, and cleaved caspase 3 proteins expression. Compared with those in cells solely overexpressing TXNDC9, the ratios of LC3II/LC3I and P62 expression were significantly greater in CRC cells overexpressing TXNDC9 treated with ICA. Moreover, there was a notable increase in the level of cleaved caspase 3 after ICA treatment, suggesting that TXNDC9 might be a crucial target for ICA‐induced programmed cell death in CRC cells, as its increase in protein expression augments its pro‐apoptotic effects. Similarly, after treatment with ICA, the expression levels of both LC3I and LC3II increased compared to those in the control group. The alterations observed in LC3I and LC3II were in line with those observed with TXNDC9 knockdown. Notably, the P62 protein level significantly decreased after TXNDC9 knockdown, in contrast to the changes observed after ICA treatment, while the level of cleaved caspase 3 decreased following TXNDC9 knockdown (Figure 5k). In addition, overexpression of TXNDC9 promoted Ica‐mediated degradation of Hsp90, while knockdown of TXNDC9 partially mitigated this effect (Figure S2, Supporting Information). We have used a pan‐caspase inhibitor (Z‐VAD‐FMK) to detect the effect of caspase activity on icaritin induced apoptosis, and the results indicated that Z‐VAD‐FMK significantly reduced apoptosis induced by icaritin (Figure S3, Supporting Information). These results suggested that in cells with low TXNDC9 expression, alternative regulatory mechanisms might be involved, resulting in decreased TXNDC9 levels that do not necessarily correlate with substantial increases in apoptosis and autophagy. However, TXNDC9 expression significantly influenced the ability of ICA to inhibit CRC cell proliferation. Thus, TXNDC9 has emerged as a critical mediator of ICA‐induced programmed cell death in CRC cells. Overall, ICA exerted its primary effects on CRC cells by modulating the interaction between HSP90 and TXNDC9, consequently reducing TXNDC9 protein levels, promoting autophagic cell death, enhancing apoptosis, and ultimately achieving its antitumor effects.

### 2.6 The Antitumor Effects and Antitumor Mechanisms of ICA In Vivo

**Figure 6:**
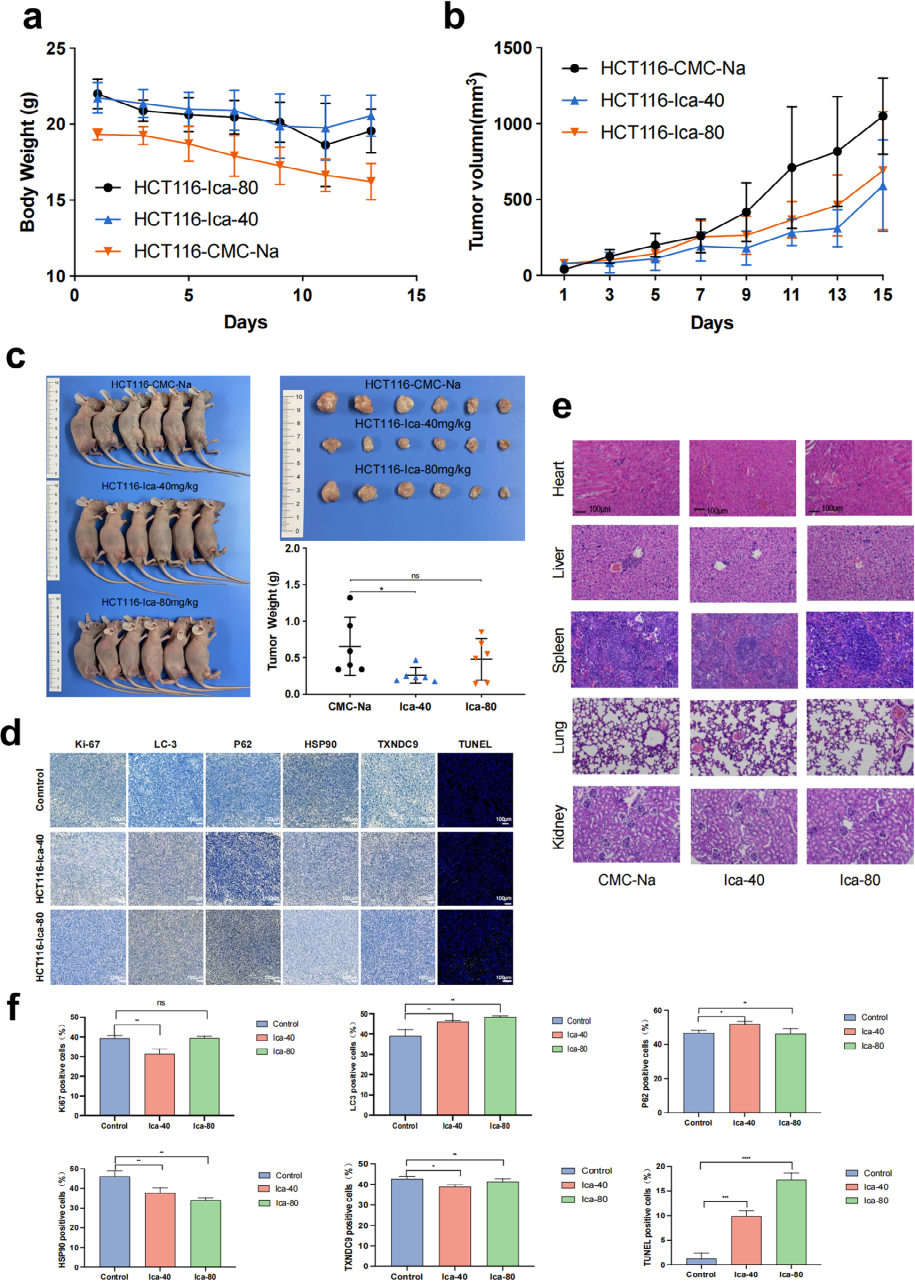
Inhibitory effects of icaritin on subcutaneous xenograft tumors in mice and molecular mechanisms. a) Changes in mouse body weight following the initiation of icaritin (ICA) treatment. b) Volumes of subcutaneous tumor tissues in mice during icaritin treatment. c) Schematic representation of mouse subcutaneous colorectal cancer xenografts. Isolated tumor tissues (top) and their weights (bottom). *P < 0.05, ns: no significance. d) Immunohistochemical analysis of the expression of various markers in mouse tumor tissues after icaritin treatment. e) H&E staining of major mouse organs at different concentrations of icaritin. f) Statistical Analysis of Immunohistochemical Markers. ns: no significance, *P < 0.05, **P < 0.01, ***P < 0.001, ****P < 0.0001.

To evaluate the in vivo anticancer effects of ICA, a subcutaneous xenograft model of CRC was established by inoculating HCT116 cells into nude mice. Once subcutaneous tumor nodules became visible and reached a size of 100 mm^2^ (calculated as length × width/2), the mice were randomly assigned to three groups: HCT116‐Ica‐40 (treated with 40 mg kg^−1^ ICA), HCT116‐Ica‐80 (treated with 80 mg kg^−1^ ICA), and a control group (HCT116‐CMC‐Na) treated with 5% carboxymethyl cellulose sodium. The changes in mouse body weight after intraperitoneal administration are presented in **Figure** **6**a, which shows no notable changes in body weight compared to the control. Figure 6b shows that ICA inhibited the growth of CRC cells. Figure 6c displays the subcutaneous tumors in the mice, with the control group exhibiting markedly larger tumor volumes than the treatment groups. The sizes and weights of the dissected tumors are displayed in Figure 6c, with average tumor weights of 0.8 ± 0.3, 0.3 ± 0.1, and 0.5 ± 0.2 g for the control group, HCT116‐Ica‐40 group, and HCT116‐Ica‐80 group, respectively. These results indicated that intraperitoneal injection of ICA effectively impeded the proliferation of CRC cells in mice. The safety of ICA was evaluated through H&E staining of mouse organs, as depicted in Figure 6e. Notably, no prominent pathological changes were observed in key organs.

Subsequently, we performed IHC staining to evaluate the expression of P62 and LC3, terminal deoxynucleotidyl transferase dUTP nick end labeling (TUNEL) phosphate buffered saline staining to detect apoptosis, the cell proliferation marker Ki‐67, and the levels of TXNDC9 and HSP90 in mouse tumor tissues. Figure 6e,f shows that treatment with ICA led to an increase in the expression of P62 and LC3 and a decrease in the expression of Ki‐67, TXNDC9, and HSP90. TUNEL staining revealed an increase, indicating an increase in the number of apoptotic cells. These findings were consistent with the results of the in vitro experiments, providing further evidence that ICA induced apoptosis and autophagic cell death in CRC cells while downregulating the expression of TXNDC9 and HSP90.

### 2.7 Construction and Characterization of the SiO_2_/Ica‐PDA‐FA Nanoparticles

**Figure 7:**
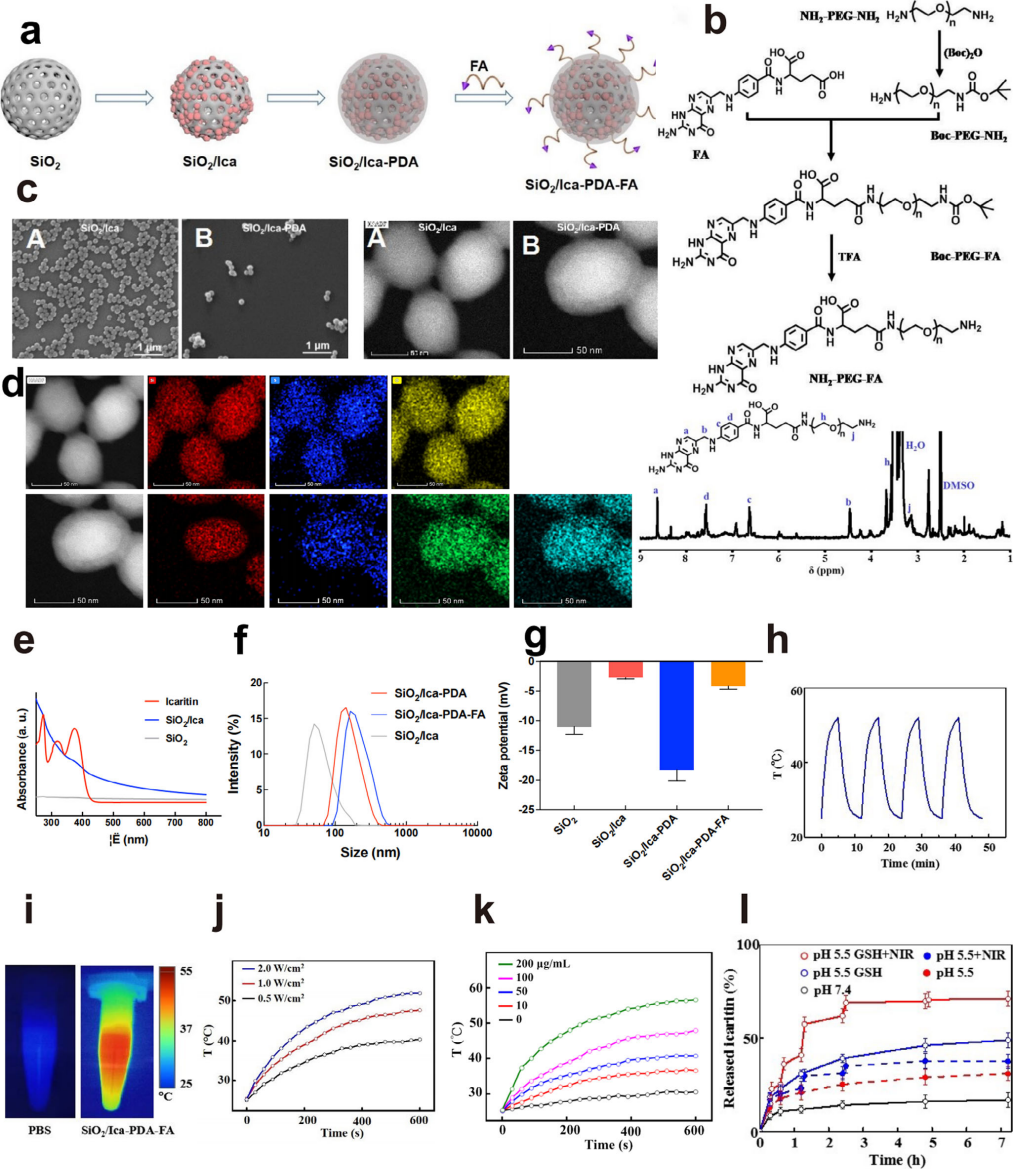
Preparation and characterization of the SiO_2_/Ica‐PDA‐FA nanoparticles. a) Schematic representation of the preparation process of the SiO_2_/Ica‐PDA‐FA NPs. b) Synthetic route (above) and nuclear magnetic resonance (NMR) spectrum (below) of the NH2‐PEG‐FA compound. c) Morphological characteristics of the SiO_2_/Ica and SiO_2_/Ica‐PDA NPs observed by SEM and TM. d) TEM images and elemental mapping analysis of the SiO_2_/Ica (above) and SiO_2_/Ica‐PDA (below) NPs. e) UV–vis spectra of the SiO_2_, icaritin, and SiO_2_/Ica. f) Particle sizes of the SiO_2_/Ica, SiO_2_/Ica‐PDA, and SiO_2_/Ica‐PDA‐FA NPs. g) Zeta potentials of the SiO_2_, SiO_2_/Ica, SiO_2_/Ica‐PDA, and SiO_2_/Ica‐PDA‐FA NPs. h) Photothermal stability of the SiO_2_/Ica‐PDA‐FA NPs. i) Photothermal images of the SiO_2_/Ica‐PDA‐FA NPs in response to NIR irradiation and PBS. j) Temperature changes of SiO_2_/Ica‐PDA‐FA NPs under different NIR irradiation powers. k) Photothermal effects at varying SiO_2_/Ica‐PDA‐FA NPs concentrations. l) Drug releases of SiO_2_/Ica‐PDA‐FA NPs.

Based on the findings of previous studies, ICA inhibits the proliferation of CRC cells, promotes autophagy and apoptosis, and suppresses HSP90 and TXNDC9 expression. Moreover, according to relevant reports, inhibiting the expression of HSP90, a type of heat shock protein, can synergistically enhance the efficacy of cancer therapies, such as chemotherapy and radiotherapy.^[^ ^32^ ^]^ Heat shock proteins play an important role in preserving protein folding and structural integrity under extreme conditions, including exposure to high temperatures, hypoxia, or drug intervention. Conventional photothermal therapies require heating tumor temperatures above 50 °C to eliminate cancer cells, yet excessively high temperatures can lead to severe side effects.^[^ ^33^ ^]^ To address the heat resistance of tumor cells, LTPTT to ablate tumors at temperatures below 45 °C was proposed. To this end, SiO_2_ nanoparticles were employed as carriers for loading ICA, and their surfaces were modified using polydopamine (PDA), which is known for its excellent biocompatibility and prolonged retention in vivo, serving as a widely accepted photothermal agent.^[^ ^34^ ^]^ Additionally, folic acid (FA) was conjugated onto the surface via Michael addition to actively target tumor tissues. This enabled the fabrication of multifunctional nanocomplexes referred to as SiO_2_/Ica‐PDA‐FA nanoparticles via the preparation process outlined in **Figure** **7**a. The synthesis route for the NH_2_‐PEG (Polyethylene glycol)‐FA compound is illustrated in Figure 7b. BOC (t‐Butyloxy carbonyl)‐protected NH_2_‐PEG‐NH_2_ was obtained by controlling the feed ratio. Following this, the carboxylic group in FA was activated and subsequently reacted with the amine group in BOC‐PEG‐NH_2_ to produce BOC‐PEG‐FA. Ultimately, the deprotection of BOC under acidic conditions yielded NH_2_‐PEG‐FA. To verify the synthesis of the NH_2_‐PEG‐FA polymer, it was dissolved in DMSO‐*d*6 for characterization through nuclear magnetic resonance (NMR) spectroscopy, and the results are shown in Figure 7B. The ^1^H‐NMR (DMSO‐*d*6, ppm, δ) signals were as follows: 8.61 (*─*NCH *─*C = N*─*), 7.53 (C_6_ H_5_CO), 6.63 (NHC_6_ H_5_), 4.42 (≡C*─*CH_2_ *─*NH*─*), and 3.5 (*─*NH*─*CH_2_ *─*CH_2_ *─*O*─*), indicating the successful synthesis of the NH_2_‐PEG‐FA polymer. Subsequently, the morphologies of SiO_2_/Ica and SiO_2_/Ica‐PDA were examined using scanning electron microscopy (SEM) and transmission electron microscopy (TEM) (Figure 7C). The SiO_2_/Ica nanoparticles exhibited a homogeneous distribution with a spherical morphology and good dispersion. Following PDA coating, the SiO_2_/Ica‐PDA nanoparticles retained their spherical form and displayed a distinct outer ring, demonstrating a consistent core–shell configuration. This observation suggested the successful encapsulation of PDA onto the SiO_2_/Ica surface while preserving its basic morphology.

TEM analysis was conducted to qualitatively determine the elemental composition of the SiO_2_/Ica and SiO_2_/Ica‐PDA nanoparticles. As shown in Figure 7d, the SiO_2_/Ica nanoparticles appeared as regularly shaped spheres that were uniformly dispersed throughout, showing the distribution of Si (red), S (blue), and O (yellow) across the nanoparticles. Moreover, the SiO_2_/Ica‐PDA nanoparticles maintained their spherical morphology, and in addition to Si, S, and O (green), they exhibited the presence of N (light blue), indicating the successful incorporation of PDA onto the surface of SiO_2_/Ica. Furthermore, the UV–visible absorption spectra (Figure 7e) exhibited the characteristic absorption peaks of ICA at 273 and 375 nm. Using these characteristic peaks, we further measured the absorption peaks of the SiO_2_/Ica nanoparticles. Even after ICA was loaded onto the SiO_2_ nanocarrier, the SiO_2_/Ica maintained characteristic absorption peaks at 273 and 375 nm. Subsequently, the dynamic light scattering (DLS) particle sizes of SiO_2_/Ica, SiO_2_/Ica‐PDA, and SiO_2_/Ica‐PDA‐FA were analyzed (Figure 7f). The hydrodynamic diameter of SiO_2_/Ica was ≈60 nm, while SiO_2_/Ica‐PDA exhibited a larger diameter of ≈115 nm, indicating size augmentation post‐PDA deposition. Introduction of the FA targeting group resulted in a slight increase in the particle size to ≈120 nm. Surface potential changes during the nanoparticle preparation process were further investigated. Figure 7g illustrates the Zeta potential of the nanoparticles, with SiO_2_ showing a potential of −11 mV. Upon ICA loading, the potential of the SiO_2_/Ica increased to −2 mV. Similarly, the PDA coating and FA grafting affected the surface potential. Compared with SiO_2_/Ica‐PDA, the final SiO_2_/Ica‐PDA‐FA nanoparticles exhibited a potential of −5 mV, enhancing the cellular uptake efficiency.

To validate the photothermal effect of the PDA‐coated nanocomplexes, photothermal images of the SiO_2_/Ica‐PDA‐FA nanoparticles were captured and compared with those of the phosphate buffered saline (PBS) group. As shown in Figure 7i, negligible temperature changes were observed in the PBS group under NIR irradiation. In contrast, SiO_2_/Ica‐PDA‐FA rapidly absorbed NIR light and elevated the suspension temperature, confirming its photothermal properties and strong photothermal effect. To achieve LTPTT, we further quantitatively analyzed the photothermal effect of the nanoparticles under various experimental conditions and concentrations (Figure 7j,k). The temperature increased with increasing NIR irradiation power, reaching ≈53, 45, and 40 °C after 5 min of irradiation at powers of 2, 1, and 0.5 W cm^−2^, respectively. Next, the SiO_2_/Ica‐PDA‐FA nanoparticles concentration influenced the photothermal effect after 5 min of 1 W cm^−2^ NIR irradiation, with temperatures of 35, 37, 45, and 54 °C observed for concentrations of 10, 50, 100, and 200 µg L^−1^, respectively. Therefore, NIR irradiation at 1 W cm^−2^ at specific concentrations (≤100 µg L^−1^) maintained temperatures below 45 °C, ensuring effective tumor ablation without adverse effects on surrounding tissues. Cycling NIR irradiation is an important method for evaluating the stability of photothermal agents. Employing this approach, we investigated the photothermal stability of SiO_2_/Ica‐PDA‐FA nanoparticles. As shown in Figure 7h, the nanoparticles underwent four consecutive cycles of NIR irradiation, with the maximum temperature remaining constant throughout. This indicated that the SiO_2_/Ica‐PDA‐FA nanoparticles maintained their photothermal stability without experiencing energy loss during treatment, underscoring their excellent photothermal performance. Furthermore, to assess the drug release behavior of the SiO_2_/Ica‐PDA‐FA nanoparticles, the tumor intracellular microenvironment was simulated using a PBS solution containing GSH (10 mm) at pH 5.5. The results, as shown in Figure 7l, revealed minimal ICA release in pH 7.4 PBS, indicating excellent stability under normal physiological conditions. Conversely, at pH 5.5, ICA release increased to 25%, suggesting enhanced release in the acidic tumor environment. The introduction of GSH further increased ICA release to 49%, which was attributed to disulfide bond cleavage accelerating drug release. NIR irradiation at pH 5.5 enhanced the release efficiency, with ICA release reaching 30%. Combined with GSH, NIR irradiation further increased the release to 70%, demonstrating a rapid response to the tumor cell microenvironment and enhanced intracellular drug delivery.

### 2.8 Uptake and Antitumor Activity of SiO_2_/Ica‐PDA‐FA Nanoparticles In Vitro

**Figure 8:**
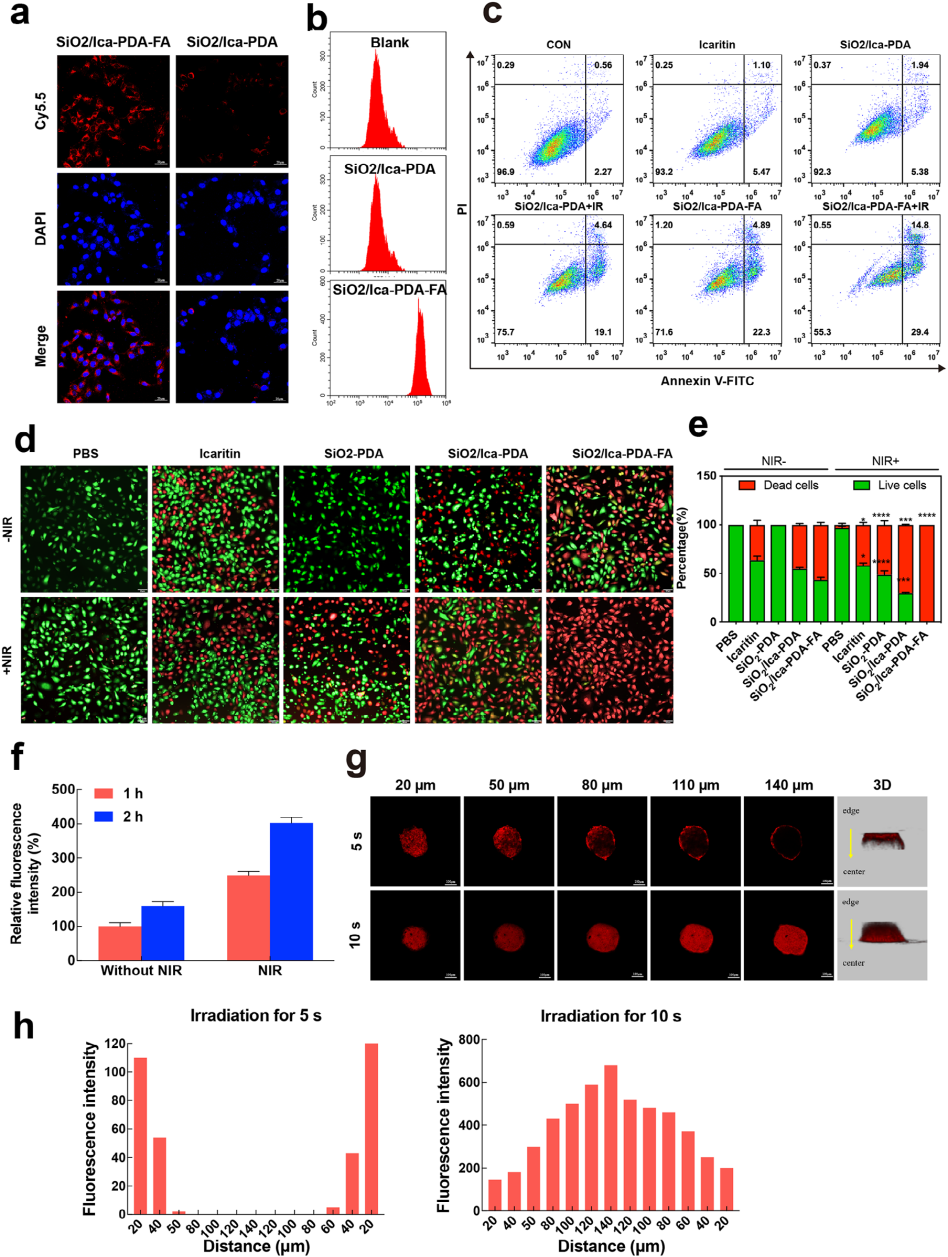
Uptake efficiency and in vitro antitumor activity of the SiO_2_/Ica‐PDA‐FA NPs. a) Confocal laser scanning microscopy (CLSM) images showing the uptake of SiO_2_/Ica‐PDA‐FA and _SiO2_/Ica‐PDA nanocomplexes by HCT116 cells. The nanocomplexes are labeled with red Cy5.5 dye, and the cell nuclei are stained blue. Scale bar: 20 µm. b) Flow cytometry analysis of the uptake efficiency of the SiO_2_/Ica‐PDA‐FA and SiO_2_/Ica‐PDA NPs by HCT116 cells. c) Flow cytometry images depicting the induction of apoptosis in HCT116 cells by nanocomplexes before and after NIR irradiation. d) Staining of colon cancer HCT116 cells for viability before and after NIR irradiation using NPs. Live cells were stained green with calcein‐AM, while dead cells were stained red with PI. Scale bar: 100 µm. e) Quantitative analysis of cell viability before and after NIR irradiation. *P < 0.05, **P < 0.01, ***P < 0.001, ****P < 0.0001. f) Impact of the duration of NIR irradiation on the permeability of the NPs. g) Z‐stack confocal fluorescence images showing the penetration of the NPs into colon cancer cell spheroids after NIR irradiation. Scale bar: 100 µm. h) Fluorescence depth profiling of nanocomplex penetration into colon cancer cell spheroids after 5 s (left) and 10 s (right) of NIR irradiation.

To validate the targeting capability of the SiO_2_/Ica‐PDA‐FA nanoparticles, we treated HCT116 cells with both SiO_2_/Ica‐PDA‐FA and SiO_2_/Ica‐PDA labeled with Cy5.5 and assessed their cellular uptake efficiency. As shown in **Figure** **8**a, cells exposed to SiO_2_/Ica‐PDA‐FA exhibited strong red fluorescence, whereas those treated with SiO_2_/Ica‐PDA showed minimal red fluorescence. These results highlighted the significant enhancement in HCT116 cell uptake facilitated by FA‐modified SiO_2_/Ica‐PDA‐FA. Similarly, flow cytometry analysis (Figure 8b) revealed markedly greater fluorescence intensity in HCT116 cells treated with SiO_2_/Ica‐PDA‐FA nanoparticles than in control cells, while cells exposed to SiO_2_/Ica‐PDA displayed slightly stronger fluorescence than did control cells. These findings suggested the limited cellular uptake of SiO_2_/Ica‐PDA by HCT116 cells and the enhanced targeting and uptake efficiency of SiO_2_/Ica‐PDA‐FA due to FA modification.

To investigate the penetration of nanoparticles into solid tumors, a CRC sphere model was established in vitro. We explored the ability of SiO_2_/Ica‐PDA‐FA to penetrate CRC cell spheres with or without NIR irradiation. Z‐stack confocal fluorescence images were captured at 20–30 µm intervals, starting from the top of the tumor sphere. As shown in Figure 8g, under NIR irradiation for 5 s, the red fluorescence within the CRC cell spheres gradually diminished, with minimal fluorescence observed beyond 110 µm and almost no fluorescence observed beyond 110 µm. This indicated that SiO_2_/Ica‐PDA‐FA nanoparticles, when irradiated for 5 s, possessed some penetration ability, although this ability was limited. Considering the impact of extended light exposure on cell viability, we increased the NIR irradiation time to 10 s. The results demonstrated that even at a depth of 140 µm within the tumor sphere, bright fluorescence was maintained. This observation was attributed to the photothermal effect, which elevated the temperature within the tumor sphere microenvironment and enhanced cell membrane fluidity. The results suggested that NIR irradiation increased the penetration ability of SiO_2_/Ica‐PDA‐FA within cell spheres, with the penetration efficiency improving as the irradiation time increased. Subsequently, we quantitatively analyzed the influence of NIR irradiation on nanocomplex penetration. As shown in Figure 8f, after NIR irradiation for 10 s, and coincubation with cell spheres for an additional hour, the fluorescence intensity within cell spheres was 2.5 times greater than that within non‐NIR‐irradiated cell spheres. After 2 h of coincubation, the fluorescence of the NIR‐irradiated cell spheres was 3.1 times greater than that of the nonirradiated cell spheres. These results indicated that NIR irradiation enhanced nanoparticle penetration into CRC cell spheres. We further conducted fluorescence depth analysis of nanoparticle penetration within CRC cell spheres (Figure 8h). Under NIR irradiation for 5 s, the penetration depth within the cell spheres was only 40 µm, while after 10 s of NIR irradiation, the penetration depth increased to 140 µm. These results further confirmed that NIR irradiation promoted SiO_2_/Ica‐PDA‐FA penetration, with longer irradiation times resulting in more significant penetration. Therefore, it was reasonable to infer that NIR facilitated the deep penetration of SiO_2_/Ica‐PDA‐FA nanoparticles into CRC tissues, which was crucial for the effectiveness of tumor treatment.

Next, we assessed apoptosis induction by SiO_2_/Ica‐PDA‐FA using Annexin V‐FITC/PI staining. As illustrated in Figure 8c, 6.57% of the ICA‐treated cells were apoptotic, while the percentage of cells in the SiO_2_/Ica‐PDA group was slightly greater, at 7.32%. This finding suggested that loading ICA into the nanoparticles could increase apoptosis levels by enhancing cellular uptake by HCT116 cells. Moreover, the SiO_2_/Ica‐PDA‐FA group had a significantly greater percentage of apoptotic cells (27.19%), indicating that FA modification facilitated the targeting ability of SiO_2_/Ica‐PDA‐FA and, in turn, intensified the apoptosis of HCT116 cells. Following NIR irradiation, the SiO_2_/Ica‐PDA+NIR group demonstrated a 23.74% apoptosis rate, while the SiO_2_/Ica‐PDA‐FA+NIR group showed a markedly elevated apoptosis rate of 44.20%. This suggested the potent inhibitory effect of SiO_2_/Ica‐PDA‐FA, particularly when combined with photothermal reactions, on the proliferation of HCT116 cells. To further assess the influence of the nanoparticles on cell viability, we stained cells subjected to free drugs or different nanoparticles (Figure 8d,e, live cells in green, deceased cells in red). Both before and after NIR irradiation, there was no notable alteration in cell viability staining in the PBS and ICA groups, indicating that the cells were not responsive to photothermal effects. However, cells treated with SiO_2_‐PDA exhibited partial cell death after light exposure, which was attributed to the photothermal toxicity caused by PDA. Following light exposure, cells treated with SiO_2_/Ica‐PDA showed additional cell death, ascribed to the cytotoxic properties of ICA, downregulation of HSP90, and cellular ablation resulting from low‐temperature photothermal effects. Notably, cells treated with SiO_2_/Ica‐PDA‐FA displayed a substantial increase in cell death, indicating that FA modification markedly amplified tumor cell uptake and cytotoxicity against tumor cells.

### 2.9 In Vivo Antitumor Effects of the SiO_2_/Ica‐PDA‐FA Nanoparticles

**Figure 9:**
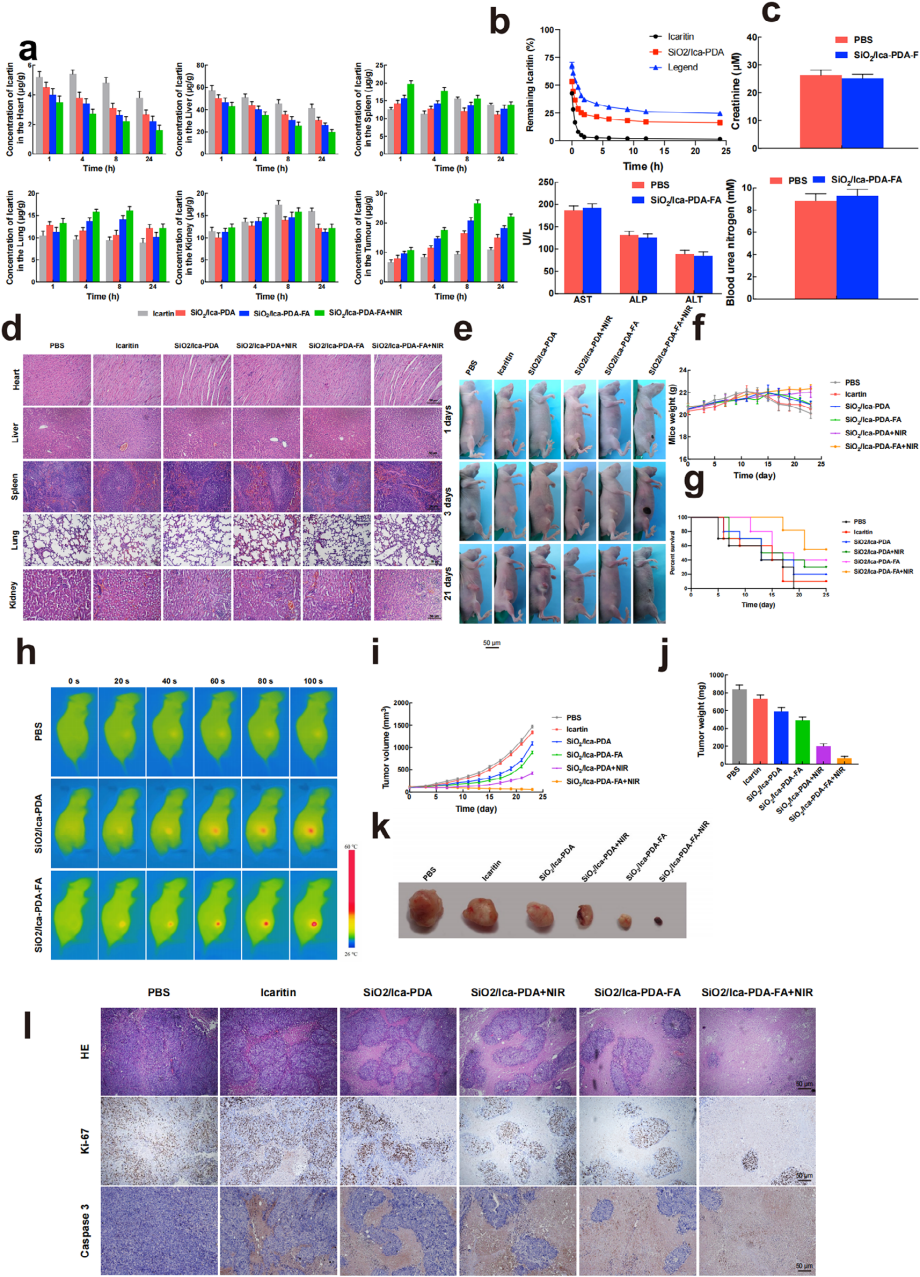
Evaluation of the in vivo antitumor effects of the SiO_2_/Ica‐PDA‐FA nanoparticles. a) Tissue distribution of SiO_2_/Ica‐PDA‐FA NPs in the heart, liver, spleen, lungs, kidneys, and tumor. b) Determination of the blood retention time. c) Hematological and biochemical analysis of liver function, blood urea nitrogen, and serum creatinine after NPs treatment. d) H&E staining of major organs in mice after NPs treatment. Scale bar: 50 µm. e) Visual changes in tumor appearance in mice after NPs treatment. f) Changes in mouse body weight during the treatment period. g) Survival curve of mice during treatment. h) Photothermal effects of the SiO2/Ica‐PDA and SiO2/Ica‐PDA‐FA NPs with different NIR irradiation times. i) Changes in tumor tissue volume in mice. j) Tumor tissue size after treatment in different groups. k) Statistical analysis of tumor changes. l) H&E and immunohistochemical staining of tumor tissues in mice from different treatment groups. Scale bar: 50 µm.

To confirm the in vivo targeting efficacy of the nanoparticles, high‐performance liquid chromatography (HPLC) was used to examine the distribution and accumulation of ICA within various organs at different time intervals. As presented in **Figure** **9**a, within 24 h of administration, the drug primarily accumulated in the liver, spleen, kidneys, and tumor tissue, indicating predominant clearance of both ICA and the nanoparticles through hepatic and renal metabolism. Compared with the other treatment groups, the SiO_2_/Ica‐PDA‐FA+NIR‐treated group exhibited a gradual increase in ICA concentration within tumor tissue from 1 to 8 h postadministration. This was attributed to the effective tumor‐targeting activity of SiO_2_/Ica‐PDA‐FA+NIR, which was facilitated by NIR irradiation and enhanced nanoparticle penetration and accumulation in the tumor, thus promoting its antitumor effect. The duration of drug circulation and retention in the body are critical factors in achieving antitumor effects. Therefore, the nanoparticle retention period in vivo was evaluated, and the results are shown in Figure 9b. Free ICA exhibited a minimal plasma concentration, which was nearly undetectable after 5 h of administration, indicating rapid clearance from the body. In contrast, the SiO_2_/Ica‐PDA and SiO_2_/Ica‐PDA‐FA groups maintained drug concentrations at 16% and 25%, respectively, even after 24 h. These findings suggested that SiO_2_/Ica‐PDA‐FA nanoparticles prolonged the circulation of ICA in the bloodstream. To assess the photothermal impact of SiO_2_/Ica‐PDA‐FA nanoparticles in mice, a mouse subcutaneous xenograft model of CRC using HCT116 cells was established. After 24 h of intravenous administration, photothermal heating images of the mice under NIR irradiation (808 nm, 120 s, 1 W cm^−2^) were captured. As shown in Figure 9h, the temperature at the tumor surface promptly increased to ≈42 °C in the. mice treated with SiO_2_/Ica‐PDA‐FA. These results suggested that FA modification enhanced nanoparticle accumulation at the tumor site.

Figure 9e illustrates the changes in subcutaneous tumor size in mice after nanoparticle treatment. Compared with those treated with SiO_2_/Ica‐PDA, mice treated with SiO_2_/Ica‐PDA‐FA showed diminished tumor volumes, underscoring the capacity of FA to bolster tumor targeting and improve antitumor efficacy. Moreover, mice treated with SiO_2_/Ica‐PDA‐FA+NIR displayed even smaller tumors than those in the SiO_2_/Ica‐PDA‐FA group, highlighting the increased antitumor impact of nanoparticle‐NIR synergy. Following a 21‐day treatment regimen, the mice were euthanized, and tumor tissues were procured for efficacy evaluation. The therapeutic effect of the SiO_2_/Ica‐PDA‐FA nanoparticles was examined by monitoring tumor volume changes over time. Figure 9k shows representative images of excised tumor tissues. The SiO_2_/Ica‐PDA‐FA+NIR group exhibited the most substantial reduction, indicating strong tumor suppression when combined with LTPTT. In the free ICA treatment group, the tumor size was smaller than that in the PBS control group, with a growth trend mirroring that of the PBS control group, suggesting a modest inhibitory effect on CRC. After SiO_2_/Ica‐PDA treatment, the tumor size decreased compared to that after free ICA treatment, possibly due to the retention of the SiO_2_/Ica‐PDA nanoparticles, which increased the drug concentration in the tumor tissue. Compared with that in the SiO_2_/Ica‐PDA group, the tumor size in the SiO_2_/Ica‐PDA‐FA treatment group slightly decreased. However, after SiO_2_/Ica‐PDA+NIR treatment, the tumor size significantly decreased compared to that after SiO_2_/Ica‐PDA‐FA treatment, accompanied by a marked decrease in tumor growth, indicating synergistic photothermal‐ICA therapy. Notably, the SiO_2_/Ica‐PDA‐FA+NIR group showed minimal tumor growth throughout the treatment period and was devoid of discernible nodules at the time of treatment, highlighting its superior tumor suppression efficacy (Figure 9H).

To gain initial insights into the mechanism underlying tumor growth inhibition by the nanoparticles in mice, tumor tissue H&E and IHC staining were performed (Figure 9l). According to the H&E images, tumor tissues treated with PBS displayed a high survival rate. After ICA treatment, necrosis of the tumor tissues was observed. Treatment with SiO_2_/Ica‐PDA, SiO_2_/Ica‐PDA+NIR, or SiO_2_/Ica‐PDA‐FA resulted in larger necrotic regions of tumor cells. Notably, treatment with SiO_2_/Ica‐PDA‐FA+NIR induced a greater degree of cell necrosis, as well as more evident shrinkage and loss of nuclear material. According to the IHC analysis for Ki‐67 and caspase 3, the PBS group displayed high cell proliferation, while tumor tissues treated with SiO_2_/Ica‐PDA, SiO_2_/Ica‐PDA+NIR, and SiO_2_/Ica‐PDA‐FA showed a gradual decrease in tumor cell proliferation and an increase in apoptotic cells. Conversely, the SiO_2_/Ica‐PDA‐FA+NIR treatment group exhibited significant inhibition of cell proliferation and a marked increase in apoptosis, reflecting outstanding in vivo antitumor efficacy.

Finally, to assess the safety of the different treatments, we examined changes in mouse body weight and survival rates during the treatment period. Figure 9f illustrates the changes in mouse body weight throughout the entire treatment duration. In the PBS, free ICA, SiO_2_/Ica‐PDA, and SiO_2_/Ica‐PDA+NIR groups, the weight of the mice initially increased, followed by a decrease as the tumors progressed. In contrast, mice treated with SiO_2_/Ica‐PDA‐FA or SiO_2_/Ica‐PDA‐FA+NIR showed a slight increase in weight throughout the treatment period, indicating the absence of significant adverse effects associated with the SiO_2_/Ica‐PDA‐FA nanoparticles, especially when combined with NIR irradiation. As depicted in Figure 9g, the median survival times of the mice in the PBS and ICA groups were 10 days and 13 days, respectively. However, treatment with SiO_2_/Ica‐PDA, SiO_2_/Ica‐PDA‐FA, and SiO_2_/Ica‐PDA+NIR led to extended survival time, with the SiO_2_/Ica‐PDA‐FA+NIR group achieving a 75% survival rate by the end of the experiment. H&E staining of heart, liver, spleen, lung, and kidney tissues from treated mice (Figure 9d) revealed no significant tissue damage associated with SiO_2_/Ica‐PDA‐FA nanoparticle treatment. Additionally, the serum levels of alanine aminotransferase (ALT), aspartate aminotransferase (AST), alkaline phosphatase (ALP), creatinine (CRE), and blood urea nitrogen (BUN) were assessed to evaluate liver and kidney function. As shown in Figure 9C, there were no alterations in the activities of ALT, AST, ALP, CRE, or BUN in the mice treated with SiO_2_/Ica‐PDA‐FA compared to those in the control group. These findings suggested that the administration of SiO_2_/Ica‐PDA‐FA nanoparticles did not induce significant liver or kidney toxicity.

## 3 Discussion and Conclusion

In this study, ICA significantly impeded the proliferation, migration, and invasion of the CRC cell lines, HCT116 and SW620 cells, in a dose‐ and time‐dependent manner and blocked the cell cycle, while displaying minimal toxicity on normal cells. Since the mechanism of ICA exert its antitumor effects in CRC is unclear yet, we explored the molecular mechanism using the quantitative proteomic analysis and revealed that ICA could significantly down‐regulate HSP90 and TXNDC9 in CRC cells, which were validated in WB analysis. Immunofluorescence colocalization and Co‐IP analysis showed that HSP90 and TXNDC9 interact directly with each other. These analyses also showed ICA down‐regulate TXNDC9 through inhibition of HSP90, possibly through direct binding to HSP90, and this effect could be rescued by HSP90 agonist tamoxifen. In addition, we found the differentially expressed proteins after ICA treatment were enriched in apoptosis and autophagy related pathways. Through fluorescence microscopy, flow cytometry, transmission electron microscopy, and WB analysis, we found that ICA suppresses cell proliferation in CRC cells by repressing autophagy to promote CRC cell apoptosis. Furthermore, through over‐expression and knock‐down of TXNDC9, we discovered that ICA regulated autophagy and apoptosis in CRC cells by attenuating the interaction between HSP90 and TXNDC9. Therefore, we revealed that in CRC, ICA regulated apoptosis and autophagy through HSP90/TXNDC9 axis, exerting its antitumor effects. The antitumor effect and mechanism of ICA was further validated in vivo through subcutaneous xenograft tumor model. Based on the inhibitory effects of ICA on HSP90, we developed the SiO_2_/Ica‐PDA‐FA nanoparticles to improve the dispersibility of ICA in aqueous solutions and maximize its pharmacological effects, achieving a dual synergistic antitumor effect.

Although ICA was only approved in hepatocellular carcinoma in by China NMPA, it exhibits potent antitumor activities across various cancer types, which include, but are not limited to liver cancer.^[^ ^35^ ^]^ In CRC, ICA exhibit its antitumor effects both in vivo and in vitro as our study shown, which were consistent with its antitumor role in CRC as reported by previous researches. Our study found that ICA induces autophagy in CRC cells, accompanied by an elevation in the ratio of LC3II to LC3I, indicative of autophagy induction in tumor cells. Intriguingly, ICA exposure leads to a concurrent upregulation of P62 expression. P62 interacts with LC3 through its LC3 interaction region (LIR) domain, thereby facilitating autophagosome formation.^[^ ^36^ ^]^ Likewise, a stable expression of the mCherry‐EGFP‐LC3B tandem construct was achieved. Our investigation revealed that following treatment with Icaritin, mCherry‐EGFP‐LC3B complex relocated to the autophagosomal membrane, presenting as numerous bright yellow fluorescent puncta under fluorescence microscopy.^[^ ^37^, ^38^ ^]^ Additionally, transmission electron microscopy unveiled the abundant formation of double‐ or multilayered membrane autophagosomes in colorectal cancer cells subjected to varying concentrations of Icaritin. The acid‐sensitive GFP is quenched in autolysosomes, while mCherry is more stable. Hence, the colocalization of both GFP and mCherry fluorescence indicates a compartment that has not fused with a lysosome, such as the phagophore or an autophagosome. Conversely, a mCherry signal without GFP corresponds to an autolysosome.^[^ ^39^ ^]^ Furthermore, in colorectal cancer cells treated with Icaritin, LC3II is degraded in the autophagosomal membranes, resulting in overlapping signals of mCherry and EGFP, thereby generating yellow puncta in the cytoplasm. This observation suggests that the autophagic flux in cells treated with Icaritin may be hindered. To further assessing the mechanism by which ICA induces autophagy in CRC cells, we systematically obstructed autophagy using different inhibitors, such as 3‐MA and HCQ, and induced autophagy using the autophagy activator rapamycin. 3‐MA inhibits autophagy by blocking autophagosome formation via the inhibition of the class III PI3K complex, which is crucial for the initiation of autophagy. By blocking the early stages of autophagy, 3‐MA effectively suppresses the initiation of autophagic processes.^[^ ^40^ ^]^ When cells were cotreated with ICA with 3‐MA, the ratio of LC3II/LC3I decreased compared to ICA treatment alone indicating that 3‐MA attenuates the promoting effect of Icaritin on autophagosome formation. HCQ functions by raising lysosomal pH and inhibiting lysosomal activity, leading to the accumulation of autophagic vesicles and impairing their degradation. This effectively blocks the final stage of autophagy, inhibiting the fusion of autophagosomes with lysosomes.^[^ ^41^ ^]^ In bladder cancer cells treated with HCQ, this inhibitory effect on autophagy is notable, primarily manifested by the impediment of autophagosome‐lysosome fusion. Consequently, the degradation pathways of p62 and LC3II are concomitantly repressed.^[^ ^42^ ^]^ inhibiting autophagy with HCQ prevented the effects induced by ICA on autophagy‐related proteins, particularly leading to further accumulation of p62. Rapamycin induces cancer cell death by inhibiting the activity of the mammalian target of rapamycin (mTOR). Rapamycin not only promotes the formation of new autophagosomes but also induces autophagosome‐lysosome fusion.^[^ ^43^ ^]^ Upon treatment with both rapamycin and ICA, a notable reduction in p62 expression levels relative to ICA treatment alone suggests that rapamycin partially mitigates the inhibitory effect of ICA on p62 degradation. This phenomenon augments the role of ICA in facilitating autophagosome formation. Based on these observations, we propose a hypothesis that Icaritin potentially promotes the conversion of cytoplasmic LC3I to membrane‐bound LC3II, during autophagosome formation. Subsequently, the selective engulfment of p62 into autophagosomes, followed by its degradation by proteases within autolysosomes, is inhibited. As a result, the entire process of autophagic flux is suppressed, ultimately leading to autophagic cell death in colorectal cancer cells.

In addition, we have conducted an in‐depth exploration of the molecular mechanisms underlying ICA‐mediated antitumor effects in CRC, and to the best of our knowledge, this is the first evidence of a direct binding between HSP90 and TXNDC9 proteins in CRC, with TXNDC9 acting as a potential client protein regulated by HSP90. Notably, our research marks the first to identify the HSP90‐TXNDC9 complex as a key antitumor target.

HSP90, a highly conserved ATP‐dependent molecular chaperone, is ubiquitously responsible for the correct folding, repairing, remodeling, and maturation of numerous client proteins. These clients include protein kinases, nuclear factors, and transcription factors, many of which are oncoproteins essential for acquiring and maintaining of the major cancer hallmarks.^[^ ^44^ ^]^ Moreover, HSP90 participates in many cellular processes, such as protein trafficking, receptor maturation, cell cycle regulation, cell survival, hormone signaling, and other signaling pathways.^[^ ^44^, ^45^ ^]^ Given its control over the maturation of oncoproteins critical in tumor development and its overexpression implicated in tumorigenesis, HSP90 has emerged as a promising target for tumor therapy. Consequently, HSP90 inhibitors have garnered significant attention for their potential as antitumor agents.^[^ ^46^ ^]^ Inhibiting HSP90 triggers the degradation of its client proteins, which are crucial for tumor growth and proliferation.

**Figure 10:**
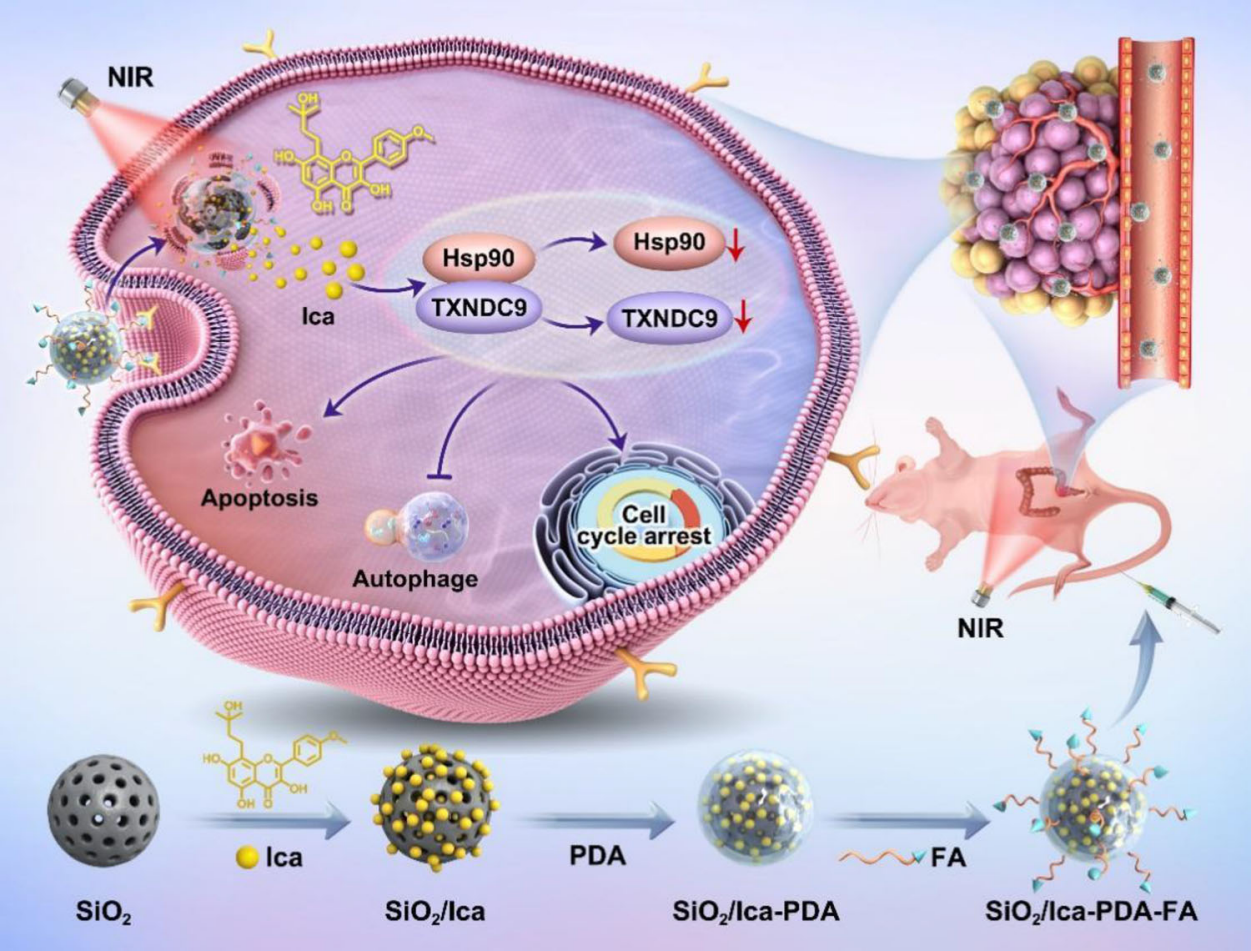
Graphical illustration of the synthesis procedure for the SiO_2_/Ica‐PDA‐FA NPs and the in vivo antitumor mechanism against CRCs. SiO_2_/Ica‐PDA‐FA NPs move to the tumor site via FA targeting, facilitating the delivery of Ica. Upon exposure to NIR (808 nm) irradiation, SiO_2_/Ica‐PDA‐FA liberates Ica, thereby exerting antitumor effects.

TXNDC9 is a member of the thioredoxin (TRX) family, which contains a series of redox proteins crucial for various biological processes and its up‐regulation has been identified as oncogenic in colorectal cancer, hepatocellular carcinoma, glioblastoma, and prostate cancer progression. Moreover, increased TXNDC9 expression has been correlated with poor prognosis in CRC.^[^ ^47^, ^48^ ^]^ Our study that ICA exerted its antitumor function through inhibiting HSP90, disrupting the HSP90‐TXNDC9 interaction, and therefore, down‐regulating TXNDC9, which were consistent with previous studies identifying TXNDC9 as oncogenic. After we over‐expressed TXNDC9, it protected CRC cells from apoptosis, and the protective effects could be eliminated by ICA. A previous study demonstrated that silencing TXNDC9 led to apoptosis induction, which was characterized by elevated levels of apoptosis‐related proteins, such as cleaved‐caspase 3, Cleaved‐caspase 8, and Cleaved‐caspase 9, as well as increased expression of autophagy markers, such as Beclin‐2 and the LC3‐II/LC3‐I ratio.^[^ ^49^ ^]^ Additionally, the suppression of TXNDC9 in A549 cells led to enhanced apoptosis, as indicated by the upregulation of Bax expression and the downregulation of Bcl‑2 expression.^[^ ^50^ ^]^ Moreover, TXNDC9 was found to protect CRC cells from oxalilpatin treatment by enhancing drug‐induced autophagy through regulation of the Nrf2 pathway.^[^ ^51^ ^]^ Our findings were consistent with these previous studies and showed the protective role of TXNDC9 through inhibiting apoptosis. Since we have found that ICA regulated autophagy and apoptosis in CRC cells through attenuating the interaction between HSP90 and TXNDC9, exerting its antitumor effects, the HSP90‐TXNDC9 axis is chosen as the target for designing a new therapy in order to best utilize the synergistic effects of ICA (**Figure** **10**).

Despite substantial efforts in developing inhibitors targeting HSP90, their clinical translation has been hindered by the protein's abundance in normal cells and its essential role in normal cellular processes. Consequently, no HSP90 inhibitor has yet been approved by the FDA.^[^ ^52^ ^]^ However, recent years have witnessed significant interest in PTT as a noninvasive strategy for tumor treatment. PTT employs near‐infrared light‐responsive materials to convert light energy into localized hyperthermia, effectively destroying tumor cells.^[^ ^19^ ^]^ Unlike traditional PTT, which often requires high temperatures (>50 °C) to induce tumor cell apoptosis or even necrosis, low‐temperature PTT (≤45 °C) has emerged as a promising alternative, minimizing the risk of heat damage to surrounding healthy tissue near the tumor and potential tumor metastasis. As an emerging strategy, offers promising potential for effective tumor ablation.^[^ ^53^ ^]^ The prevalent approach involves loading HSP90 inhibitor into photothermal materials to effectively suppress HSP90 expression within tumors. This strategy aims to surmount tumor heat resistance and induce mild‐temperature heating effects.^[^ ^54^ ^]^ Folate receptors are highly expressed in varied malignant tumors and only little expressed in normal tissues, folic acid‐modified nanoparticles could selectively target cancerous cells through receptor‐mediated endocytosis.^[^ ^55^ ^]^ In our study, we capitalized on the ability of ICA to inhibit HSP90 and its client protein TXNDC9, coupled with its low bioavailability when administered alone. We developed tumor‐targeted SiO2/Ica‐PDA‐FA nanocomposites and utilized NIR low‐temperature photothermal therapy for effective treatment for colorectal cancer. Characterization of the SiO2/Ica‐PDA‐FA nanocomplexes revealed uniform particle size of 120 nm, a zeta potential of −5 mV and a core–shell structure with excellent photothermal stability. These nanocomplexes exhibited effective cellular uptake by HCT116 cells, suppressed tumor growth under NIR irradiation, and penetrated CRC tumor spheroids. In vivo experiments further confirmed the gradual reduction in subcutaneous tumor in mice, confirming favorable biocompatibility and safety profiles.

In conclusion, these findings illuminate the multifaceted mechanisms by which ICA exerts its antitumor effects in CRC cells, encompassing apoptosis induction, autophagy modulation, and interference with specific TXNDC9‐HSP90 interactions. Additionally, the study extended to in vivo assessments, affirming the safety and efficacy of ICA in a mouse xenograft model. The formulation of SiO_2_/Ica‐PDA‐FA nanocomplexes, complemented by low‐temperature photothermal therapy, displayed remarkable targeting and deep penetration into CRC tissues, underlining its potential as a treatment strategy. Furthermore, the nanocomplexes demonstrated biosafety, as evidenced by the absence of significant changes in body weight or organ damage in treated mice. This suggests that ICA, in conjunction with SiO_2_/Ica‐PDA‐FA nanocomplexes and low‐temperature photothermal therapy, holds promise for CRC treatment.

## 4 Experimental Section

### Cells and Animals

Two human CRC cell lines (HCT116 and SW620), and a normal  HIEC were obtained from the American Type Culture Collection (ATCC, Manassas, VA). HCT116 cells were cultured in RPMI 1640 medium, and SW620 and HIEC cells were maintained in Dulbecco's modified Eagle's medium (DMEM). Each culture medium contained 10% fetal bovine serum (FBS) and 1% penicillin/streptomycin (Gibco) and was maintained at 37 °C in a humidified incubator with 5% CO_2_.

BALB/c nude mice (weighing 18–22 g, 6–8 weeks old) were procured from Huafukang Bioscience Co., Inc. (Beijing, China) and housed under specific pathogen‐free (SPF) conditions. These conditions included a humidity level of 60% ± 10%, a controlled temperature of 22 ± 2 °C, and a 12/12‐h light/dark cycle at the Animal Center of Sichuan University. The animal protocols used were approved by the ethics committee of West China Hospital, Sichuan University, and all the experimental procedures were conducted in accordance with the guidelines of the Institutional Animal Care and Use Committee of the Model Animal Research Center.

### Transfection of siRNA and Overexpressed Plasmid

The siRNAs targeting TXNDC9 and negative controls (NCs) were procured from Shanghai GenePharma Co., Ltd. Cell culture involved seeding cells in 6‐well plates when they reached 30%–40% confluency and were maintained in opti‐MEM medium (Gibco). siRNA Transfection of (5 nmol per well) was performed using Lipofectamine 2000 Transfection reagent (Invitrogen, USA) according to the manufacturer's protocols. Following transfection, the cells were incubated for 6 h before the medium was replaced with complete medium, after which the cells were further incubated for 24 h

293T cells were transfected with a recombinant lentivirus carrying a TXNDC9 overexpression plasmid (VectorBuilder, Guangzhou, China). The TXNDC9 protein and GFP were expressed under the control of the pCMV and eEF promoters, respectively. The plasmids pMD2. G and psPAX2 were acquired from Addgene (Addgene plasmid # 12259; Addgene plasmid # 12260). The cell media was changed 3 h before transfection. After reaching 60%–70% confluency, 293T cells were transfected with the plasmids. Following the protocol, transfection was carried out using a lentiviral transfer vector (pLV [Exp]‐EGFP‐Puro‐hTXNDC9 or pLV [Exp]‐EGFP‐Puro‐empty) in conjunction with the plasmids pMD2. G and psPAX2, employing the calcium phosphate transfection method (Beyotime, Shanghai, China). Twenty‐four hours after transfection, the supernatant was collected and concentrated. CRC cells were then cultured in a 6‐well plate and when they reached 70% confluency, they were exposed to the concentrated lentivirus in the presence of polybrene. After 24 h, the process was repeated to induce secondary infection. Subsequently, media containing 5 µg mL^−1^ puromycin was added to eliminate uninfected control cells until complete eradication. Transfection efficiency was determined through qPCR and Western blot analyses. Finally, the cells were harvested and prepared for subsequent assays.

### Cell Proliferation Assay

The cell proliferation assay was used to evaluate the effectiveness of ICA in inhibiting cell growth using a cell counting kit‐8 (CCK8, Sigma, 57360‐69‐7). Briefly, cells were plated in 96‐well plates at a density of 3–4 × 10^3^ cells per well during the logarithmic growth phase (in six replicate wells) and treated with ICA (0–60 µm) for 24 or 48 h. Next, 10 µL of CCK‐8 reagent was added to each well, and the plates were incubated at 37 °C for 1–2 h. Subsequently, the absorbance at 450 nm was quantified for each well using a microplate reader (Molecular Devices, CMax Plus). For the colony formation assay, cells were seeded in 6‐well plates at a density of 1–1.5 × 10^3^ cells per well and treated with ICA (0.625–10 µm) for 7–10 days. The incubation continued until macroscopic colonies became visible in the dishes, and the medium was changed as needed. Thereafter, the cell supernatant was removed, and the colonies were carefully rinsed with ice‐cold PBS, followed by fixation in 4% paraformaldehyde for 10 min. After fixation, the colonies were stained with 0.5% crystal violet. After the excess dye was rinsed off with running water, the colonies were enumerated.

### Cell Wound Scratch, Migration, and Invasion Assays

Cells were seeded in 6‐well plates at a density of ≈3 × 10^5^ cells per well and allowed to reach 80% confluency. A sterile 200‐µL pipette tip was then used to gently create a scratch across the center of the monolayer. Afterward, the cells were washed with PBS to eliminate cellular debris and then incubated in medium supplemented with ICA (15 and 30 µm) or vehicle. Images were captured at 0 and 24 h after the initiation of the scratch, and the wound gaps were quantified using ImageJ software.

For the migration assay, the cells were suspended in serum‐free media at a concentration of 5 × 10^5^ mL^−1^ and then transferred to the upper chamber of the an 8‐µm pore size membrane (Corning, USA). For the invasion assay, the upper transwell membranes were precoated with Matrigel (BD bioscience) before seeding cells in serum‐free media into the chambers (≈2 × 10^5^ mL^−1^). Then, 600 µL of culture media containing 10% FBS was added to the lower chambers. Following a 48‐h incubation period at 37 °C, any remaining cells on the upper membrane were gently removed with a cotton tip. Following migration or invasion through the membrane, the cells were stained with 0.5% crystal violet after being fixed with 4% paraformaldehyde for 10–15 min and subsequently counted under a microscope. The cells were divided into three groups and assessed in triplicate.

### Cell Cycle

HCT116 and SW620 cells were initially plated in 6‑well plates at a concentration of 2 × 10^5^ mL^−1^. Subsequently, the cells were exposed to the required concentration of ICA (0, 7.5, 15, or 30 µm) for 24 h. After treatment, the cells were collected, rinsed with cold PBS, and fixed in procooled 75% ethanol overnight at 4 °C. Following fixation, the samples were treated with RNase A (50 µg mL^−1^) in a water bath at 37 °C for 2 h and then stained with propidium iodide (PI, 50 µg mL^−1^) for 1 h at 4 °C in the dark. Flow cytometry analysis was conducted using a BD flow cytometer (BD Biosciences, USA), and the data were analyzed with FlowJo software. Each experimental condition was replicated three times.

### Cell Apoptosis Analysis

For the Hoechst staining assay, cells were prepared and seeded according to the *cell cycle* analysis protocol. Following exposure to different concentrations of ICA (0, 7.5, 15, or 30 µm), the cells were fixed with 4% paraformaldehyde (PFA) for 15 min and subsequently stained with Hoechst 33258 dye at a concentration of 10 µg mL^−1^ for 10 min. Then, the cells were rinsed with PBS and imaged immediately using fluorescence microscopy. For quantitative assessment of apoptosis, cells were harvested, washed with PBS, and suspended in binding Bbuffer. Annexin V‐FITC staining was performed for 10–15 min, followed by PI staining for 5 min, and all steps were carried out in the dark. The samples were then detected using flow cytometry, and the data were processed with FlowJo software for further analysis.

### Transfection of the mCherry‐GFP‐LC3 Plasmid

Cells cultured in 6‐well plates at a density of 1 × 10^3^ mL^−1^ were transfected with the mRFP‐EGFP‐LC3 plasmid and then incubated for 48 h. Transfection efficiencies exceeding 85% were confirmed, and the cells were then grown on laser confocal culture dishes for 24 h before treatment with ICA (15 µm) for an additional 24 h. Following treatment, the cells were stained with Hoechst for 15 min after being fixed in 4% paraformaldehyde for 10 min. The mRFP‐EGFP‐LC3 fluorescent signal was observed using Zeiss LSM 780 confocal microscopy (Carl Zeiss) to analyze autophagic flux.

### Transmission Electron Microscopy

Cells in the logarithmic growth phase were exposed to ICA at concentrations of 15 and 30 µm for 24 h. After treatment, the cells were fixed in 2.5% glutaraldehyde solution and 0.1% OsO_4_ in PBS at 4 °C overnight. Following three washes with PBS, the samples were dehydrated in an ethanol series (50%, 70%, 80%, 90%, and 100% ethanol) for 15–20 min each. Subsequently, the samples were transferred to absolute acetone and embedded in pure epoxy resin. Ultrathin sections were then prepared and stained with uranyl acetate and lead citrate, before being subjected to examination under a transmission electron microscope.

### Western Blot Analysis

CRCs were treated with 30 µm Icaritin and/or 1 mm 3‐MA (or 5 µm HCQ, 1 µm Rapa) for investigating autophagy‐related proteins. Additionally, CRCs were treated with 30 µm Icaritin, 10 µm HSP90 inhibitor Geldanamycin, or 30 µm HSP90 activator Tamoxifen (Tamox). Cellular proteins were isolated using ice‐cold RIPA buffer supplemented with PMSF. Briefly, sample protein concentrations were determined using the Pierce Rapid Gold BCA Protein Assay Kit (Thermo Fisher Scientific, Inc.) in accordance with the manufacturer's guidelines. Subsequently, the protein samples were combined with 5 × loading buffer and heated at 100 °C for 5 min to denature them. After denaturation, 40 µg of each protein sample was resolved on 7.5%–15% SDS‐PAGE gels and subsequently transferred to polyvinylidene fluoride (PVDF) membranes. Post‐transfer, the membranes were blocked with a 5% nonfat milk solution for 1–2 h at room temperature. Next, the membranes were subjected to three 10‐min washes with TBST (TBS containing 0.1% Tween‑20) buffer and incubated overnight at 4 °C with the following specific primary antibodies: anti‐caspase 8 (CST, 9746S), anti‐cleaved caspase 8 (CST, 9492S), anti‐caspase 3 (Gene Tex, GTX110543), anti‐cleaved caspase 3 (CST, 9664T), anti‐caspase 9 (CST, 9508S), anti‐cleaved caspase 9 (CST, 7273S), anti‐PARP(Abcam, ab32138), anti‐cleaved PARP (Abcam, ab32561), anti‐Bcl‐2 (Proteintech, 12789‐1‐AP), anti‐Bax (Proteintech, 50599‐2‐Ig), anti‐cytochrome c (Proteintech, 10993‐1‐AP), anti‐Fas (Abcam, ab82419), anti‐GAPDH (Proteintech, 60004‐1‐Ig), anti‐P62 (Proteintech, 18420‐1‐AP), anti‐LC3B (Sigma, L7543), anti‐CD44 (Proteintech, 60224‐1‐Ig), anti‐Cathepsin D (Proteintech, 21327‐1‐AP), anti‐CHK1(Proteintech, 25887‐1‐AP), anti‐CyclinD1 (Proteintech, 60186‐1‐Ig), anti‐CDK4 (Proteintech, 66950‐1‐Ig), anti‐PDCD4(Proteintech, 12587‐1‐AP), anti‐MEK2(Proteintech, 67410‐1‐Ig), anti‐AKT2 (Proteintech, 17609‐1‐Ig), anti‐TXNDC9 (Abcam, ab185959), anti‐HSP90 (Gene Tex, GTX109753). Following primary antibody incubation, the membranes were washed three times with TBST and then treated with HRP‐conjugated secondary antibodies (anti‐rabbit or anti‐mouse) from Proteintech (SA00001‐3/2) at a dilution of 1:100 000 for 1 h at room temperature. The protein bands were visualized using Immobilon ECL Ultra Western HRP Substrate (Merck KGaA), captured with a ChemiDoc System (Bio‑Rad Laboratories, Inc.), and quantified with ImageJ software.

### Tandem Mass Tagging (TMT)‐Based Quantitative Proteomics

Protein quantification followed the same protocol as for Western blot analysis. For protein digestion, 200 µg of the protein sample was suspended in lysis buffer containing 8 m urea and 100 mm dithiothreitol (DTT) and then immediately boiled for 5 min. After boiling, the samples were mixed with urea buffer, transferred to ultrafiltration tubes, and centrifuged at 13 000 rpm for 30 min to remove the supernatant. The denatured samples were alkylated by adding iodoacetamide (IAA)‐urea buffer and left to react in the dark at room temperature for 30 min. Afterward, the samples were suspended in 40 µL of 0.1 m triethylammonium bicarbonate (TEAB) before incubation overnight at 37 °C with 4 µg of trypsin, and the peptides were collected as filtrates. Next, the tryptic peptides from each sample were labeled using a TMT kit (Thermo Fisher Scientific) according to the manufacturer's instructions. Following labeling, the peptides were separated using an HPLC system with mobile phases A (10 mm NH_4_HCO_3_ and 5% acetonitrile) and B (10 mm NH_4_HCO_3_ and 85% acetonitrile, pH 10). High‐pH reverse phase chromatography was used to fractionate the peptides into 15 fractions. For LC‐MS/MS analysis, the peptides were reconstituted in 0.1% formic acid and analyzed by a 90‐min gradient on an EASY‐nLC 1000 UPLC system coupled with a Q Exactive HF‐X mass spectrometer. The detection method was set to positive ion mode with a 90‐min analysis duration and a parent ion scanning range of 350–1800 m/z. The resolution of the first‐level mass spectrometer was set to 70 000 at m/z 200. The top 10 most abundant precursor ions in the full scan were selected and fragmented by higher‐energy collisional dissociation. The resolution for the second‐level mass spectrometry was set to 17 500 at m/z 200.

The LC‐MS/MS data were analyzed using Proteome Discoverer software (version 2.2, Thermo Fisher, USA). For each TMT experiment, the raw data files from all fractions were combined, and MS2 spectra were searched against the Uniprot database (Uniprot_HomoSapiens_20367_20200226) using the Sequest HT and Mascot (v2.6.0) search engines. The false discovery rate (FDR) thresholds were set at 1% for proteins, peptides, and peptide spectral matches (PSMs). Proteins were categorized into biological process (BP), cellular component (CC), and molecular function (MF) categories using Gene Ontology (GO), and the KEGG database was utilized for protein pathway annotation.

### Immunohistochemistry (IHC) and Immunofluorescence (IF)

The protein expression of key genes was assessed via IHC. Tissue slides were formalin‐fixed and paraffin‐embedded. Following deparaffinization and rehydration of histological sections, endogenous peroxidase activity was quenched by treatment with 3% H_2_O_2_ for 10 min. The sections were subjected to antigen retrieval by incubating them in sodium citrate buffer (10 mm, pH 6.0) for 30 min at 100 °C. Subsequently, they were blocked with 2% bovine serum albumin (BSA) for 30 min at room temperature (RT). Thereafter, the sections were incubated with primary antibodies (anti‐TXNDC9 and anti‐HSP90) for 1 h at RT, followed by incubation with the corresponding secondary antibodies and 3′,3′‐diaminobenzidine (DAB) chromogen for staining. The sections were ultimately counterstained with hematoxylin, dehydrated, and mounted using Vectamount for subsequent microscopic examination.

For IF, cells were cultured on cell chamber slides and subjected to the same treatments. The cells were fixed with ice‐cold 4% paraformaldehyde for 10 min, followed by two PBS washes. Then, the cells were permeabilized with 0.2% Triton X‐100 in PBS for 10 min and blocked with 2% BSA for 30 min at room temperature. Subsequently, primary antibodies were applied to the cells overnight at 4 °C. After washing, the cells were treated with fluorochrome‐conjugated secondary antibodies for 1 h at RT, followed by counterstaining with DAPI. Finally, the slides were mounted and visualized using a Leica confocal microscope.

### Coimmunoprecipitation (Co‐IP)

Cells were treated as described for Western blot analysis, and lysed with precooled IP buffer on ice for 30 min, and precipitated by centrifugation. The protein concentration was assessed using a BCA kit. A/G magnetic beads were incubated with primary antibodies (IgG, TXNDC9, and HSP90) for 1 h on a rocking platform. Cell lysates were then captured using protein‐conjugated beads overnight at 4 °C. The beads were collected, boiled in 5 × loading buffer for 5 min, subjected to SDS‐PAGE, transferred onto a PVDF membrane, and then analyzed.

### Quantitative PCR (qPCR) Analysis

According to the manufacturer's instructions, total RNA was extracted using an RNA extraction reagent kit (Foregene, Chengdu, China). RNA samples were then quantified, and 1 µg of total RNA was used for cDNA synthesis with a PrimeScript RT reagent kit with gDNA Eraser (Takara Bio Inc.). qPCR was performed with TB Green Premix Ex Taq II (Takara Bio Inc.) on a CFX96 Touch Real Time PCR instrument (Bio‐Rad). The sequences of the primers used for TXNDC9 were as follows: forward, 5′‐CATTCCCACACTAGCACTGC‐3′; and reverse, 5′‐GGTTCTGAAATGGTGGCTCC‐3′; and for GAPDH, forward, 5′‐TCAAGAAGGTGGTGAAGCAGG‐3′; reverse, 5′‐TCAAAGGTGGAGGAGTGGGT‐3′. The expression levels of genes were normalized to those of GAPDH, and the relative expression levels were calculated using the ∆Ct method.

### Preparation of the SiO_2_/Ica‐PDA‐FA Nanoparticles

Mesoporous silica nanoparticles (MSNs) were synthesized according to a slightly modified protocol as previously described.^[^ ^56^ ^]^ Briefly, *N*‐cetyl‐*N*,*N*,*N*‐trimethylammonium bromide (cetyl trimethyl ammonium bromide (CTAB), Merck) surfactant was dissolved in 110 mL of distilled water, 10 mL of anhydrous ethanol, and 0.875 mL of ammonia (2 m) at 80 °C. Subsequently, 1.75 mL of tetraethyl orthosilicate (TEOS) and an equivalent volume of bis(triethoxysilyl propyl) disulfide (BTSPD) were added under vigorous stirring. After stirring at 80 °C for 5 h, the resulting white precipitate was harvested via centrifugation at 18 000 rpm for 20 min. Subsequently, it was washed thrice with deionized water and anhydrous ethanol, respectively, and dissolved in acidic methanol solution (50 mL of methanol and 1 mL of HCl) for 8 h by reflux extraction to remove excess reactants. ICA (2 mg) was dissolved in 10 mL of ethanol containing SiO_2_ and then added to the synthesized silica sol under stirring for 24 h. The resulting SiO_2_/Ica nanocomplexes were collected by centrifugation, washed with ethanol, and lyophilized.

### Preparation of Polydopamine (PDA)‐Modified SiO_2_/Ica

PDA (100 mg) was dispersed in 100 mL of Tris‐HCl buffer (10 mm, pH 8.5), followed by the addition of 100 mg of SiO_2_/Ica and stirring for 12 h at RT. The “core–shell” SiO_2_/Ica‐PDA nanoparticles were obtained by centrifugation and then washed three times with deionized water.

### Folic Acid‐Modified SiO_2_/Ica‐PDA Nanoparticles

First, 60 mL of 1,4‐dioxane was used to dissolve 0.206 g of (Boc)_2_O, and the solution was then added dropwise to an ice bath containing NH_2_‐PEG‐NH_2_ (2 g) solubilized in 30 mL of NaHCO_3_ (0.1 mol L^−1^), followed by stirring for 18 h at RT. Once the reaction was finished, the solution pH was adjusted to 7.0 with HCl (2 m), and the solvent was evaporated under vacuum. The resulting product was then extracted with dichloromethane, yielding a yellow oily substance named BOC‐PEG‐NH_2_. Next, 1 g of FA was dissolved in a solution containing 2 g of EDC and 1.2 g of NHS in PBS, followed by stirring for 4 h at RT. The FA solution was combined with BOC‐PEG‐NH_2_, and the mixture was stirred at room temperature for 24 h. After extraction with dichloromethane and removal of the solvent by evaporation, an intermediate named BOC‐PEG‐FA was obtained. Then, 1 g of BOC‐PEG‐FA was mixed with 10 mL of dichloromethane, and trifluoroacetic acid (TFA) was added, followed by magnetic stirring for 2 h to deprotect the Boc group. After evaporation of the TFA solvent, the resulting solid was NH2‐PEG‐FA. Finally, NH2‐PEG‐FA (1 mg), EDC (2 mg), and NHS (1.2 mg) were mixed in PBS and stirred for 4 h at RT. The solution was then added dropwise to SiO2/Ica‐PDA in distilled water (10 mL). After 24 h of stirring at room temperature, the SiO_2_/Ica‐PDA‐FA nanoparticles were collected, washed with PBS, and dried.

### Characterization of SiO_2_/Ica‐PDA‐FA Nanoparticles

Nuclear magnetic resonance (NMR) of NH2‐PEG‐FA was performed by dissolving the NH2‐PEG‐FA polymer in DMSO‐*d*6. Monodispersed tetramethylsilane standards were used for calibration. After dilution with deionized water, the particle size and Zeta potential of the SiO_2_/Ica, SiO_2_/Ica‐PDA, and SiO_2_/Ica‐PDA‐FA nanoparticles were measured via dynamic light scattering (DLS) using a Malvern Nano ZS instrument. A laser with a wavelength of 633 nm was employed, and each sample was measured at 25 °C. In addition, the size and morphology of the nanoparticles in aqueous solution were determined by scanning electron microscopy (SEM) and TEM, respectively. NPs were stained with phosphotungstic acid, dispersed on a copper mesh, and air‐dried. SEM images were captured using a Hitachi S‐4800 instrument (Hitachi, Japan), while TEM observations were conducted after drying the samples on copper microgrids.

### Drug Release

SiO_2_/Ica‐PDA‐FA was subjected to drug release assays under NIR illumination or in the absence of NIR. Specifically, the nanoparticles were incubated with PBS solution at pH 5.5 and pH 7.4, with or without glutathione (GSH), utilizing dialysis bags with a molecular weight cutoff of 3.5 kDa under continuous shaking at 37 °C. These conditions were chosen to mimic diverse physiological environments. At the scheduled time points, an external sample of 1 mL was collected, and an equal volume of fresh buffer solution was added. The drug concentration in the release media was analyzed by a UV–visible spectrophotometer.

### Photothermal Effect and Stability

The photothermal properties of SiO_2_/Ica‐PDA‐FA were monitored with an infrared thermal imaging system. Various concentrations of nanoparticles (10, 50, 100, and 200 µg mL^−1^) suspended in PBS were irradiated with an 808 nm NIR laser at various power densities (0.5, 1, 2 W cm^−2^). in Real‐time monitoring of temperature changes in the sample suspensions was conducted with an infrared thermal imaging camera. In addition, the stability of the photothermal effect was investigated by subjecting nanoparticles at a constant concentration (100 µg mL^−1^) to irradiation with an 880 nm laser for 5 min; this process was repeated four times using the same methodology.

### Cell Uptake

HCT116 cells were cultured in 6‐well plates and incubated with Cy5.5‐labeled SiO_2_/Ica‐PDA and SiO_2_/Ica‐PDA‐FA for 24 h. After that, the cells were washed with PBS, fixed with 4% PFA for 30 min, and stained with DAPI for 10 min in the absence of light. Images of the cells were captured and analyzed using confocal laser scanning microscopy.

### Tumor Spheroid Infiltration Assay

To prevent cell adhesion, the 96‐well plate was pretreated with 80 µL of 1.5% w/v agarose gel. HCT116 cells (2 × 10^3^ per well) were seeded and cultured for 15 days to allow for spheroid formation, and the medium was replaced every other day. After full spheroid formation, the tumor spheroids were cocultured with SiO_2_/Ica‐PDA‐FA nanoparticles for 24 h, and then irradiated with NIR. Afterward, the cell spheroids were washed with PBS, fixed with 4% PFA, and then transferred to a chambered coverslip for confocal microscopy analysis.

### In Vivo Antitumor Effects

BALB/c nude mice were subcutaneously injected with HCT116 cells (4 × 10^6^) suspended in a Matrigel matrix into the right flanks to establish a tumor model. Once the tumor nodules reached ≈100 mm^3^ (calculated as 1/2 × length × width^2^), the mice were randomly divided into three treatment groups (*n* = 6): 0.5% CMC‐Na vehicle, Icaritin at 40 mg kg^−1^, and Icaritin at 80 mg kg^−1^. Intraperitoneal (i.p.) administration was performed for 2 days for 14 days. Tumor size and animal weight were monitored every 2 days until sacrifice. Upon study completion, xenografted mice were euthanized under anesthesia induced by intraperitoneal injection of sodium pentobarbital. Subcutaneous colon adenocarcinoma tissues were weighted and fixed in 4% PFA for histopathology and IHC analysis, while organs (spleen, heart, liver, kidney, and lung) were collected for H&E staining.

To assess the in vivo targeting of the SiO_2_/Ica‐PDA‐FA nanoparticles, a distribution experiment was conducted. Tumor‐bearing mice were segregated into four distinct groups: icaritin, SiO_2_/Ica‐PDA, SiO_2_/Ica‐PDA‐FA, and SiO_2_/Ica‐PDA‐FA+NIR (808 nm, 1 W cm^−2^; 120 s). The drugs were intravenously (i.v.) administered at an equivalent dosage of Ica (10 mg kg^−1^). At 1, 4, 8, and 24 h postinjection, the mice were euthanized, and the liver, heart, lung, spleen, kidney, and tumor tissues were harvested. Tissue samples were ground with anhydrous ethanol, and centrifuged at 3500 rpm for 5 min at 4 °C, after which the supernatant was subjected to HPLC analysis.

To evaluate the antitumor effects of SiO_2_/Ica‐PDA‐FA nanoparticles, tumor‐bearing mice were randomly assigned to different groups and received intravenous injections of PBS vehicle (*n* = 6), icaritin (10 mg kg^−1^), SiO_2_/Ica‐PDA, SiO_2_/Ica‐PDA+NIR, SiO_2_/Ica‐PDA‐FA, or SiO_2_/Ica‐PDA‐FA+NIR. Twenty‐four and 48 h postadministration, the SiO_2_/Ica‐PDA +NIR and SiO_2_/Ica‐PDA‐FA+NIR groups were irradiated with an 808 nm laser (1 W cm^−2^) for 120 s. Temperature changes were documented utilizing an infrared thermal imaging camera (Fluke Ti27).

### Histological Analysis

Tissue specimens from tumors or mice were fixed, and 5 µm thick paraffin‐embedded tissue sections were prepared. Routine hematoxylin and eosin (H&E) staining was performed to visualize the nuclei and cytoplasm.

### Statistical Analyses

Prism GraphPad 8.0 and SPSS 28.0 were used for the statistical analysis of the experimental data. Significant differences between samples were determined using *t*‐tests and one‐way ANOVA. ImageJ software was used for image analysis. Each experiment was conducted in triplicate, and the results are presented as the mean ± standard deviation (mean ± SD).

## Conflict of Interest

The authors declare no conflict of interest.

## Author Contributions

D.H. and S.C. contributed equally to this manuscript. The manuscript was written through contributions of all authors. All authors have given approval to the final version of the manuscript.

## Supporting information



Supporting Information

## Data Availability

The data could be accessed from the corresponding author upon reasonable request.
