# Supplementary material for: Icaritin Represses Autophagy to Promote Colorectal Cancer Cell Apoptosis and Sensitized Low‐Temperature Photothermal Therapy via Targeting HSP90‐TXNDC9 Interactions
Source: Adv Sci (Weinh). 2025 Apr 4;12(20):2412953. doi: 10.1002/advs.202412953 (PMC12120733; doi:10.1002/advs.202412953)
Supplement: Supplementary file 1 — Supporting Information [file ADVS-12-2412953-s001.docx]

**Supporting information**

**Icaritin represses autophagy to promote colorectal cancer cell apoptosis and sensitized low-temperature photothermal therapy via targeting HSP90-TXNDC9 interactions**

Dan He^1,2,†^, Siliang Chen^1,3,†^, Xiaoyun Wang^1,3,*^, Xiang Wen^1,3^, Changyang Gong,^1^ Lei Liu^1,*^, Gu He^1,3,4,*^

*^1^ Department of Dermatology & Venerology and Division of Head & Neck Tumor Multimodality Treatment, Cancer Center, West China Hospital, Sichuan University, Chengdu 610041, China*

*^2^ Department of Oncology, The Second Affiliated Hospital of Chengdu Medical College, China National Nuclear Corporation 416 Hospital, Chengdu 610053, China*

*^3^ Laboratory of Dermatology, Clinical Institute of Inflammation and Immunology, Frontiers Science Center for Disease-related Molecular Network, State Key Laboratory of Biotherapy, West China Hospital, Sichuan University, Chengdu 610041, China*

*^4^ Institute of Precision Drug Innovation and Cancer Center, the Second Hospital of Dalian Medical University, Dalian 116023, China*

**Corresponding Authors**

Xiaoyun Wang: wxyun08109@163.com

Lei Liu: liuleihx@gmail.com

Gu He: Email: hegu@scu.edu.cn.


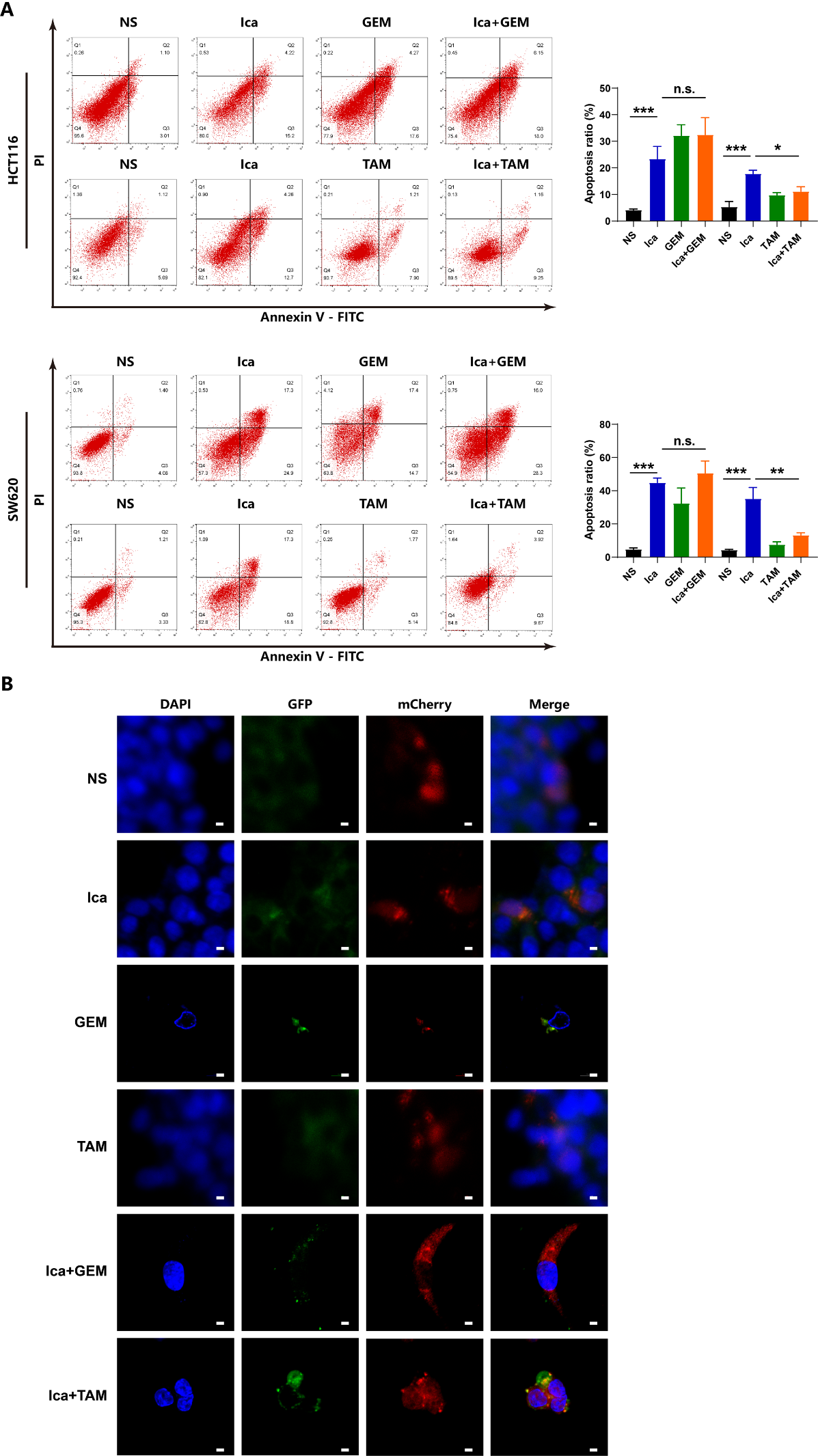


**Figure S1.** (A) Flowcytometry analysis of cellular apoptosis in colorectal cancer cells by Annexin V-FITC/PI dual-staining, the cells were treated by icaritin alone or in combination of Hsp90 inhibitor GEM (Geldanamycin) or Hsp90 agonist TAM (Tamoxifen); (B) Immunofluorescence analysis of autophagosomes and autolysosomes in HCT116 colorectal cancer cells by transfected mCherry-GFP-LC3 plasmid, the cells were treated by icaritin alone or in combination of Hsp90 inhibitor GEM (Geldanamycin) or Hsp90 agonist TAM (Tamoxifen). Scale Bar: 6μm.


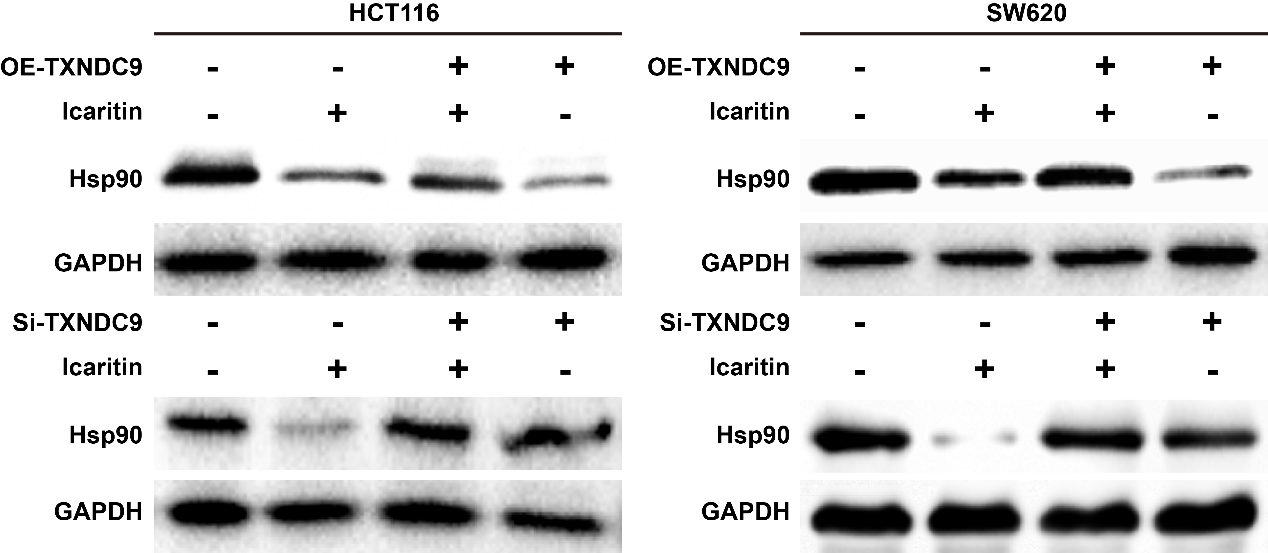


**Figure S2.** WB analysis of Hsp90 protein expression in colorectal cancer cells treated with icaritin and TXNDC9 overexpression or RNA interference.


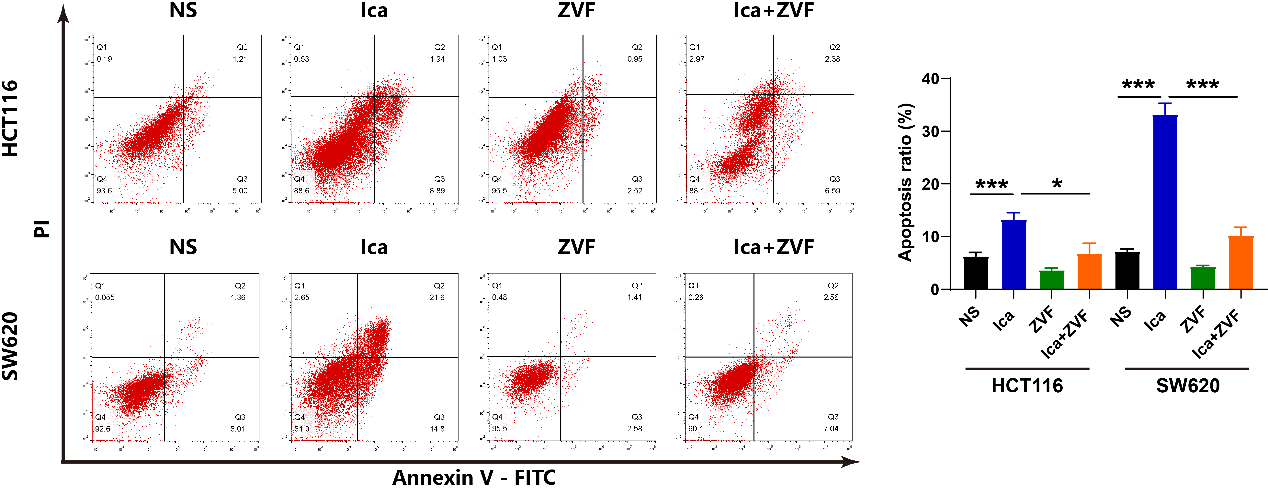


**Figure S3.** Flowcytometry analysis of cellular apoptosis in colorectal cancer cells by Annexin V-FITC/PI dual-staining, the cells were treated by icaritin alone or in combination of pan-caspase inhibitor ZVF (Z-VAD-Fmk).
